# Measurement and modelling of deep sea sediment plumes and implications for deep sea mining

**DOI:** 10.1038/s41598-020-61837-y

**Published:** 2020-03-19

**Authors:** Jeremy Spearman, Jonathan Taylor, Neil Crossouard, Alan Cooper, Michael Turnbull, Andrew Manning, Mark Lee, Bramley Murton

**Affiliations:** 1 HR Wallingford, Howbery Park, Wallingford, Oxfordshire, OX10 8BA UK; 2 National Oceanography Centre, European Way, Southampton, SO14 3ZH UK

**Keywords:** Ocean sciences, Physical oceanography

## Abstract

Deep sea mining concerns the extraction of poly-metallic nodules, cobalt-rich crusts and sulphide deposits from the ocean floor. The exploitation of these resources will result in adverse ecological effects arising from the direct removal of the substrate and, potentially, from the formation of sediment plumes that could result in deposition of fine sediment on sensitive species or entrainment of sediment, chemicals and nutrients into over-lying waters. Hence, identifying the behaviour of deep-sea sediment plumes is important in designing mining operations that are ecologically acceptable. Here, we present the results of novel *in situ* deep sea plume experiments undertaken on the Tropic seamount, 300 nautical miles SSW of the Canary Islands. These plume experiments were accompanied by hydrographic and oceanographic field surveys and supported by detailed numerical modelling and high resolution video settling velocity measurements of the *in situ* sediment undertaken in the laboratory. The plume experiments involved the controlled formation of benthic sediment plumes and measurement of the plume sediment concentration at a specially designed lander placed at set distances from the plume origin. The experiments were used as the basis for validation of a numerical dispersion model, which was then used to predict the dispersion of plumes generated by full-scale mining. The results highlight that the extent of dispersion of benthic sediment plumes, resulting from mining operations, is significantly reduced by the effects of flocculation, background turbidity and internal tides. These considerations must be taken into account when evaluating the impact and extent of benthic sediment plumes.

## Introduction

### Background

There is increasing global concern over the long-term availability of secure and adequate supplies of critical raw materials, known as E-tech elements, which have an essential contribution to emerging ‘green’ technologies^1^. These E-tech elements are present (in different concentrations and combinations) in poly-metallic nodules on the ocean floor and cobalt-rich crusts on seamounts^2^. Additionally, seafloor massive sulphide deposits contain economically attractive concentrations of a variety of minerals including copper, gold, silver and zinc^3^. Plans to extract these minerals involve a seafloor harvester, creating sediment disturbance through its motion across the sea bed, cutting of the substrate, collection of the minerals, discharge of uneconomic sediment after processing and, potentially, discharge of over-burden covering buried deposits^4–7^. Such operations will remove substrate (and any ecology bound to, or buried within, the substrate), and also generate turbidity plumes that may result in deposition of sediment onto sensitive species in the near to far-field and/or entrainment of sediment, nutrients and chemicals into over-lying waters.

A review of previous sediment resuspension experiments^8^ describes the various deep sea plume experiments undertaken to date^9–13^. These experiments mainly consisted of towed harrow-like disturbers, dragged across the abyssal plain in the hope they would reproduce the effects of mining, with post-event evaluation using box-coring and sediment traps. However, these approaches have been focused towards identifying changes in substrate rather than plume behaviour, and are insufficient to provide suitable data sets for validating dispersion models, particularly those involving the mining of Cobalt-rich crusts.

### Previous modelling studies of deep sea mining plumes

Modelling studies of deep sea mining (e.g.^14–17^) have, to date, concentrated on the effects of collection of nodules from the abyssal plain, or of mining of massive sulphides at Solwara (ref. ^4^) with little or no studies involving the mining of crusts^18^. In the main these studies, which include near-bed and mid-water plumes, have noted the importance of flocculation in reducing the extent of any dispersion but did not assess this effect, instead using settling velocity values based on *in situ* particle size (mean settling velocities of 0.01–0.1 mm/s). As a result, the settling velocities were based on the sediment particle size distribution and were much lower (in general) than the values measured from our present study that looked at the actual behaviour of particles suspended in the water column. The modelling studies on the abyssal plain have, in particular, emphasised the long distances over which individual (fine) sediment particles, disturbed by mining, would travel before re-settling onto the bed, but did not assess the distance over which any increase in plume concentration would become small compared to the natural background concentration, which is the more relevant concern^19^. Such studies have led to considerable speculation about extensive and widespread impacts of deep sea mining activities (e.g.^18,20–22^), which may not be merited.

More recently^23^ Gillard *et al*. modelled the impact of Collector tests in the eastern section of the German license area of the CCZ. The modelling simulated the (near-bed) plume dispersion resulting from 4 days of collector tests using settling velocities derived from laboratory measurements of *in situ* sediment (deriving settling speeds in the range 0.08–4.0 mm/s). While the study did not investigate the spatial dispersion of the plume in the water column, it found that the vast majority of released sediment settled to the bed within a day of the cessation of mining, and all of this deposition occurred within 9 km even under extreme ocean eddy conditions. As in the present study the Gillard *et al*. study indicates the importance of flocculation in the subsequent dispersion of the benthic plume.

As well as the type of mining, the local soil conditions and the technology of the collector, the characteristics of tailings plumes released in the water column depend heavily on the design of the on-board processing that will be applied to separate the ore from the uneconomic tailings. This can have a significant effect on the nature of the tailings plume. For instance, estimates of tailings dry solids discharges from mining of massive sulphides have varied from 2 kg/s^4^ to 70 kg/s^24^. Evaluating the effects of tailing discharges without a detailed mining design is therefore prone to considerable uncertainty. For this reason the focus of this paper has concentrated on the nature and dispersion of benthic plumes arising from mining.

#### Focus of the present study

**Figure 1:**
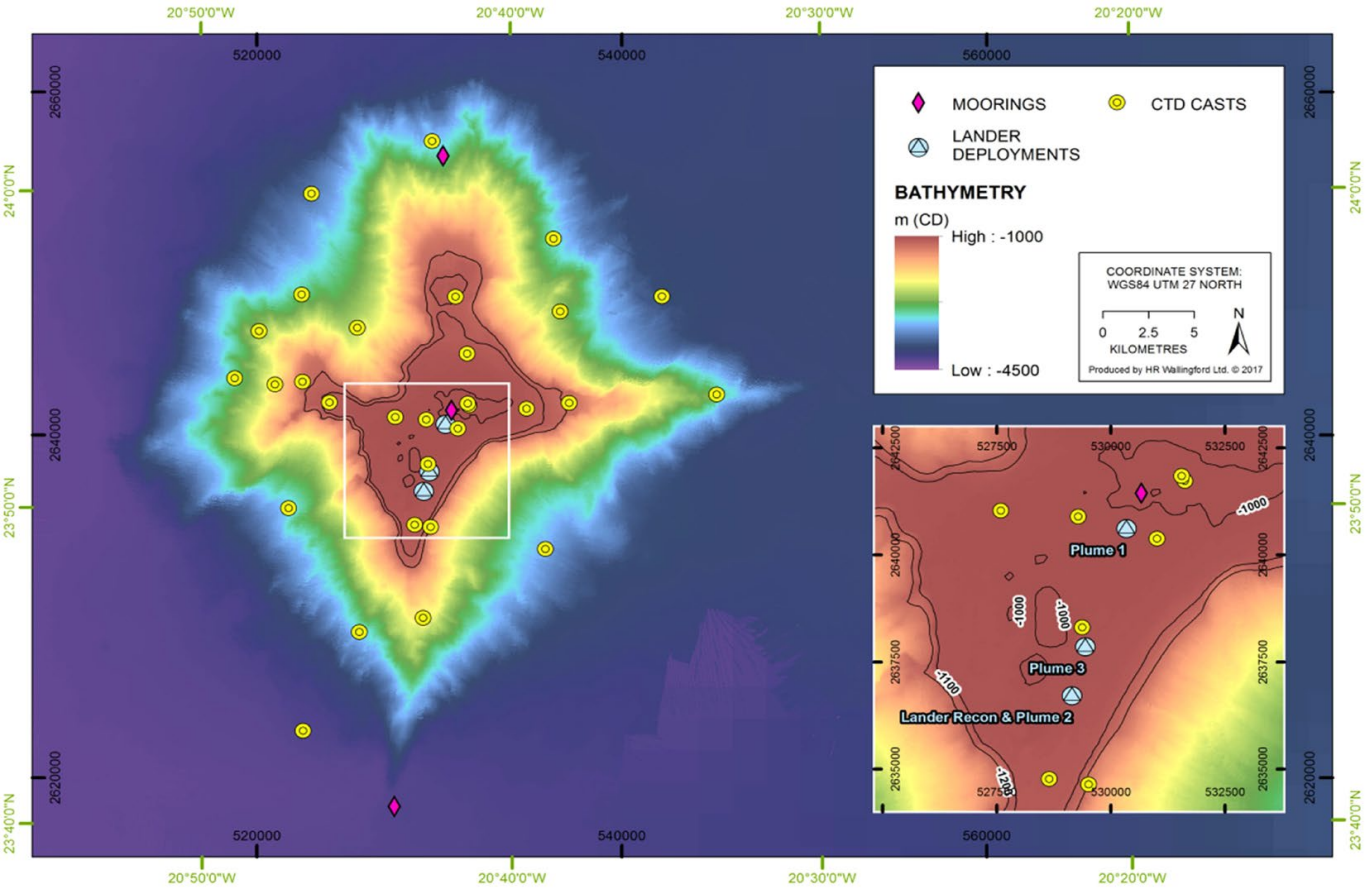
Tropic Seamount and locations of moorings, CTD casts and the three plume experiments at the lander deployments.

**Figure 2:**
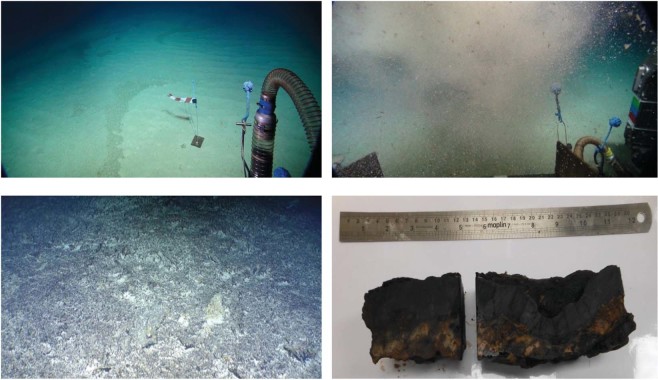
(Bottom left) Crust pavement. (Bottom right) Crust sample from Tropic Seamount. (Top left) Sandy sediment used for the *in situ* plume experiments. (Top right) Sediment plume seen from the ROV.

The studies described in this paper test the hypothesis that sediment plumes generated at the sea bed by deep sea mining will be limited in spatial extent owing to a combination of effects of sediment flocculation and dispersion to levels below the natural variation in the background suspended sediment signal. The hypothesis is investigated in detailed studies associated with the mining of Cobalt-rich crusts but, as discussed later, many of the key considerations also arise for the mining of nodules and massive sulphides. The studies identify the behaviour of deep sea benthic plumes on the Tropic Seamount through a combination of field experiments, laboratory studies and numerical modelling. This seamount lies approximately 300 nautical miles SSW of the Canary Islands and has a star-shaped planform with a width of approximately 40 km at the base and about 15 km at the crest (Fig. 1). Its summit rises 3000 m from the abyssal floor to a depth of ~1000 m where it forms a flat plateau partially covered by mobile sediment deposits of silty sand, with the remainder being covered by cobalt-rich crusts, occurring mainly as pavements (Fig. 2). Cobalt-rich crust also occurs on the flanks as pavements and as loose blocks, associated with slope collapse^25^.

The *in situ* field experiments initiated and tracked sediment plumes using a combination of a specially designed benthic lander (equipped with four optical backscatter sensors - OBS: at 0.5, 1.0, 1.3 and 1.5 m above the bed) and an upward looking acoustic Doppler current profiler - ADCP), a remotely operated underwater vehicle (ROV) and an autonomous underwater vehicle (AUV). In total, 19 successful plume experiments were carried out, at three locations in the central and southern parts of the seamount summit. Plumes were generated by the ROV at distances 25, 50 and 100 m from the benthic lander. Plumes were generated using a pump attached to the ROV, releasing sediment sucked from the bed into the water immediately above the vehicle with the flux monitored by the pump’s flow-rate and the volume of sediment excavated from the seabed. Positioning of the ROV, benthic lander and AUV were carefully choreographed on the basis of hydrodynamic model forecasting (verified by earlier benthic lander deployments), and streamers placed on the seabed at the time of the experiment (see methodology section for details).

This ensured the maximum likelihood of the sensors encountering the centre of the plumes down-stream from the ROV. The field experiments were supported by laboratory settling tests, made on *in situ* sediments and seawater samples recovered from the field sites by ROV, using LabSFLOC-2 high resolution video-imaging technology^26^. These were conducted to understand the potential for flocculation of the sediment and crust material and to identify the resulting distribution of settling velocities for both materials. Detailed numerical modelling, informed by current and salinity profiling and data gathered from three deep-ocean moorings deployed around the seamount, was undertaken to reproduce the sediment plume transport during the field experiments and to provide the hydrodynamic information to drive the sediment plume model.

## Results

### Hydrodynamic modelling

**Figure 3:**
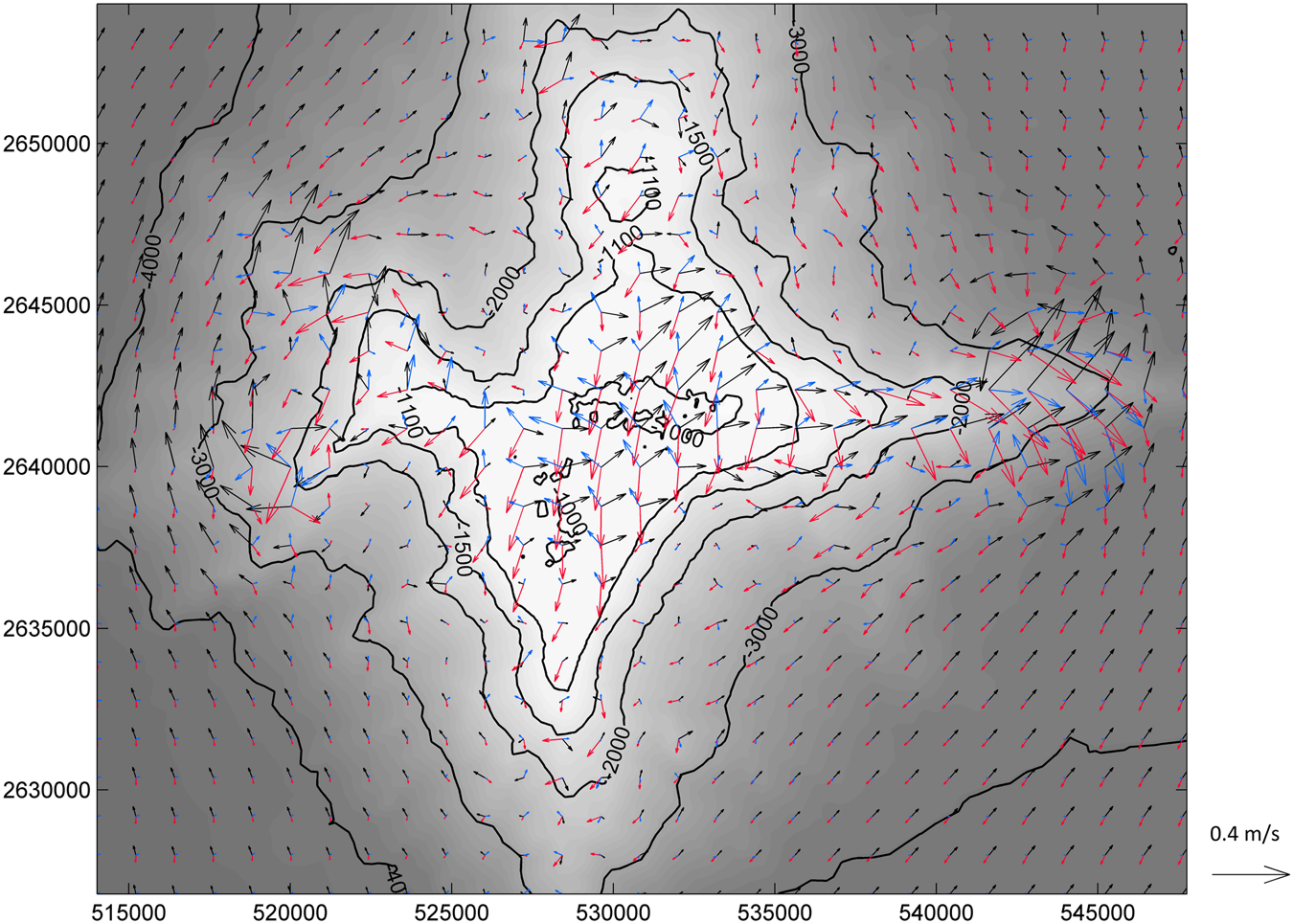
Snapshots of currents 10 m above the bed in the vicinity of the seamount on a spring tide at HW (black), HW + 4 hours (red) and HW + 8 hours (blue).

**Figure 4:**
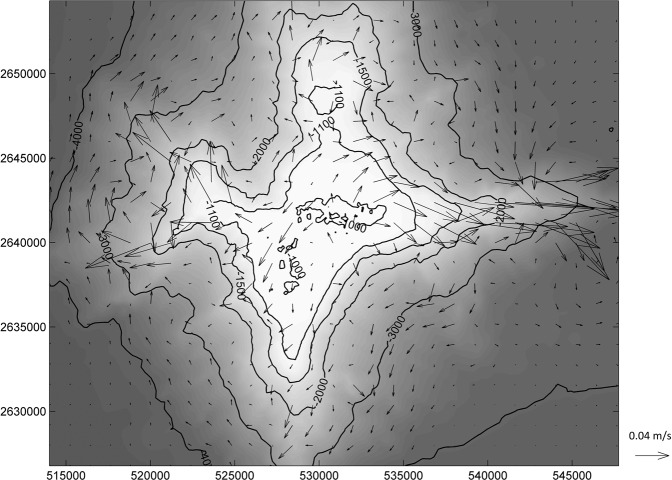
Residual currents 10 m above the bed in the vicinity of the seamount over the period 14 Nov to 21 Nov 2016.

The movement of water at the summit (approx. 1000 m below sea level) of the Tropic Seamount was observed to be dominated by internal tide-generated currents that rotate in an anti-cyclonic direction about the seamount’s centre. Snapshots of modelled spring-tide currents 10 m above the bed are shown in Fig. 3, illustrating the rotational nature of the flow. Hydrodynamic modelling identified that current speeds varied up to 0.3 m/s, with the highest values on the east and west “spurs” of the seamount, and that a weak Taylor cap exists around the seamount, close to the sea bed^27^. Figure 4 shows the residual currents 10 m above the bed over a 7 day run in November 2016 which illustrates a weak anticyclonic current around the summit of the seamount. The strength of the Taylor cap effect retaining particles close to the bed can be assisted by three-dimensional tidal flow^28,29^ and the significance of these effects is a combination of seamount morphology, ocean stratification and the incident flow^29,30^.

The tidal variation was observed to be semi-diurnal but (as also noted in refs. ^31,32^ amongst others), transfer of energy via sub-harmonic resonance from the semi-diurnal internal tidal harmonic also resulted in significant diurnal components of current flow, even though there is minimal external diurnal influence. This phenomenon only occurs equator-ward of the 28.8°S and 28.8°N latitudes where the M1 diurnal frequency exceeds the magnitude of the local Coriolis parameter, $f=2\Omega sin\varphi$, (where $\Omega$ is the earth’s angular velocity, and *φ* is the latitude), allowing the diurnal component to propagate as a free internal wave^32^.

During the field experiments, *in situ* measurements confirmed that the local currents varied between 0.03 m/s and 0.2 m/s but typically were around 0.1 m/s.

**Figure 5:**
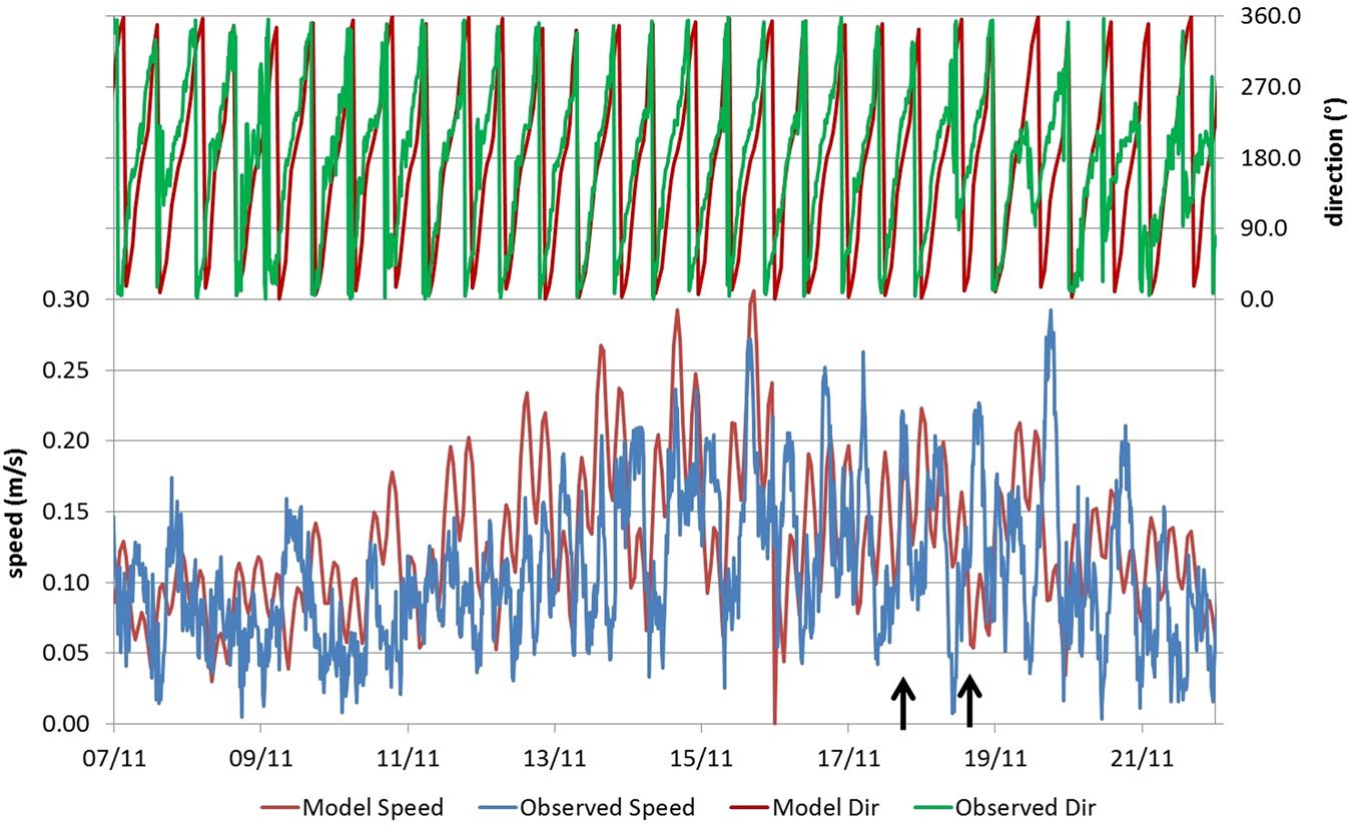
Observed current magnitude (blue) and direction (green) and predicted current magnitude (red) and direction (red) over a fortnight in November 2016. The timing of plume experiments on 17 and 18 November are indicated by black arrows.

A comparison of the measured and predicted current velocities 10 m above the seamount summit during a fourteen day period including the plume experiments on 17 and 18 November is shown in Fig. 5 (for location see Fig. 1).

### Sediment and crust characteristics

The sediment used for the plume experiments (Fig. 2a) was predominantly “muddy sand” (on average 1% gravel, 84% sand and 15% silt/clay) and pelagic in origin^25^. Pterapod shells were clearly visible in the plumes created in the plume experiments (Fig. 2b). The cobalt-rich crust on the seamount occurs mainly as pavement (Fig. 2c) with thicknesses (Fig. 2d) of 3 cm (on average) and up to 16 cm. Typically, the crust has bulk and dry densities of 1950 kg/m^3^ and 1740 kg/m^3^, respectively^25^. The crust material investigated in this study was derived from the debris left from cutting of samples recovered by the ROV, and was virtually all less than 63 µm in diameter.

### Plume observations

Out of the 36 plume experiments undertaken, 19 plume experiments registered a plume at the lander and produced good data and the resulting observations are summarised in Table 1. Typically, the suspended sediment concentration measured at the lander was a few mg/l. During the passing of the plume, the ADCP registered backscatter at heights of 20–30 m – which gives an estimate of the vertical extent of the plume. On average the pump generated a source term of around 0.2 kg/s but instantaneously the pump achieved a release rate an order of magnitude higher than this. AUV measurements of turbidity found that the plume reduced to suspended sediment concentrations similar in order to the background concentrations (order 10 µg/l) at a distance of around 1 km. Fourier analysis of back-scatter signals from a 4-day deployment of the benthic lander-mounted ADCP, in preparation for the first plume experiment, identified turbidity signals at approximately quarter-diurnal, semi-diurnal and diurnal frequencies indicating significant tidal components of the turbidity signal.

**Table 1:** Summary of plume measurements.

Nominal range to lander	Average currents during ROV plume generation		Approx. Time taken for plume to reach lander (seconds)	Average SPM Concentration During Plume event (mg/l)*		
	Speed (m/s)	Direction (Deg N)		Bottom OBS (0.5 m above bed)	Mid OBS (1.0 m above bed)	Top OBS (1.5 m above bed)
***Location 1***						
25 m	0.07	198	332	4.4	3.5	3.1
25 m	0.09	194	243	9.7	6.4	5.1
50 m	0.11	200	432	2.6	1.8	1.4
100 m transect	0.13	227	757	1.0	0.8	0.8
***Location 2***						
25 m transect	0.11	135	200	1.4	1.4	1.8
25 m	0.05	145	467	1.3	1.5	1.6
25 m	0.03	156	680	0.4	0.5	0.6
50 m transect	0.14	177	360	6.0	5.3	5.6
50 m	0.11	179	445	1.7	1.3	1.8
50 m	0.18	184	267	6.3	4.3	3.8
50 m	0.15	182	300	5.0	4.2	4.2
100 m transect	0.12	206	840	3.9	3.3	3.4
100 m	0.13	194	730	2.7	1.9	1.8
***Location 3***						
25 m	0.09	176	307	0.3	0.4	0.5
25 m	0.11	162	271	1.0	1.1	1.5
50 m transect	0.10	163	497	4.2	3.4	3.2
50 m	0.11	162	458	0.6	0.5	0.5
50 m	0.08	161	561	1.0	0.8	0.7
100 m transect	0.09	189	1023	1.1	1.2	1.3

*Results from the OBS mounted at 1.3 m above the bed are not presented as this OBS was optimised for capturing suspended sediment concentrations of at least two orders of magnitude above the typical concentrations recorded.

#### Settling velocity properties of seabed sediment and crust debris

Laboratory tests, using LabSFloc-2 high resolution video-technology^33^, were carried out on the settling characteristics of both the sediment and the debris arising from cutting of the crust (the latter acting as a proxy for the fine sediment fraction of mining debris). Tests were carried out using *in situ* samples of seawater from near to the seamount surface for suspended sediment concentrations of 20 and 100 mg/l. A summary of the experiment results is given in Table 2. The tests showed that flocculation occurs in both the sediment and in the crust debris (indicated by floc density being significantly lower than that of solid sand particles, around 2600 kg/m^3^, or of crust aggregates, around 1750 kg/m^3^). Overall mean settling velocities (weighted by mass, Ws) were 10–11 mm/s for the sediment and around 9 mm/s for the crust debris.

**Table 2:** Summary of settling velocity measurements (values weighted by mass).

Sediment concentration (mg/l)	Microflocs (<160 μm) or macroflocs (>160 μm)	Diameter (μm)	Effective dry density (kg/m^3^)	Ws (mm/s)	Proportion of the total mass (%)	Flocs sampled
*Sediment*						
20 mg/l	Microflocs	127	793	7.5	38	19
	Macroflocs	197	651	13.8	62	20
100 mg/l	Microflocs	114	669	5.7	37	101
	Macroflocs	205	581	12.3	63	97
*Crust debris*						
20 mg/l	Microflocs	76	452	7.4	55	78
	Macroflocs	207	223	11.5	45	7
100 mg/l	Microflocs	114	547	6.6	50	277
	Macroflocs	177	551	12.2	50	44

The settling velocity was found to be similar for both suspension concentrations for both microflocs and macroflocs (here defined as flocs with diameters less than and greater than 160 µm, after ref. ^34^). Gillard *et al*.^23^ also observed rapid flocculation of fine to coarse silts from the Clarion Clipperton seabed surface in artificial seawater, but resulting in lower overall settling velocities than those in Table 2. The higher settling velocities observed in our experiments are considered to result from the flocculation enhancing role of the extra-cellular polymers and bacteria^35–38^ occurring in real sea water, (including the deep sea^30^), the electrostatic properties of crust particles^39^ and the ability of fine sediment (in combination with biogenic material) to flocculate with larger sand-sized particles^40–42^.

Additional settling experiments, recording the change in concentration in a glass jar over time, were also conducted to identify the mass contribution of particles not detected by the LabSFLOC-2 experiments. These tests (Table 3) showed that, while most of the settling behaviour corresponded to the rapid settling observed in the LabsFloc-2 experiments, around 9% of the sediment and 3.5% of the crust debris formed very small flocs (predominantly < 40 µm in diameter) that settled much more slowly (<0.2 mm/s), and which were not reliably detected by the video technique.

**Table 3:** Summary of jar settling measurements (values weighted by mass).

Sediment type	Nominal initial concentration (mg/l)	Time elapsed (mins)	Percentage of sediment left in jar after elapsed time (%)
Sediment	100	2	12.5
		20	8.5
		180	9
Crust	100	2	22.5
		20	3.5
		180	2.5

Based on the information from the LabSFLOC-2 and jar tests, the distribution of size fractions and their respective characteristic settling velocities was derived (Table 4). The laboratory measurements needed to be adjusted to obtain the corresponding deep-sea values, owing to the effect of water temperature on viscosity^43^.

**Table 4:** Characteristic distribution of floc sizes and settling velocities.

Particle size (μm)	Sediment			Crust debris		
	% mass	Characteristic settling velocity (mm/s)		% mass	Characteristic settling velocity (mm/s)	
		Laboratory experiments	Adjusted to deep sea temperature		Laboratory experiments	Adjusted to deep sea temperature
>160	54.25	13 or more	12 or more	37	12	11
120–160	23.25	7	6.5	20	10	9.2
40–120	10.5	2.6	2.4	20.5	3	2.7
<40	3.75	0.2	0.18	19	0.5	0.46
	0.0	0.025	0.023	1	0.025	0.023
	8.75	0.004	0.0036	2.5	0.004	0.0036

### Numerical modelling of sediment plume experiments

**Figure 6:**
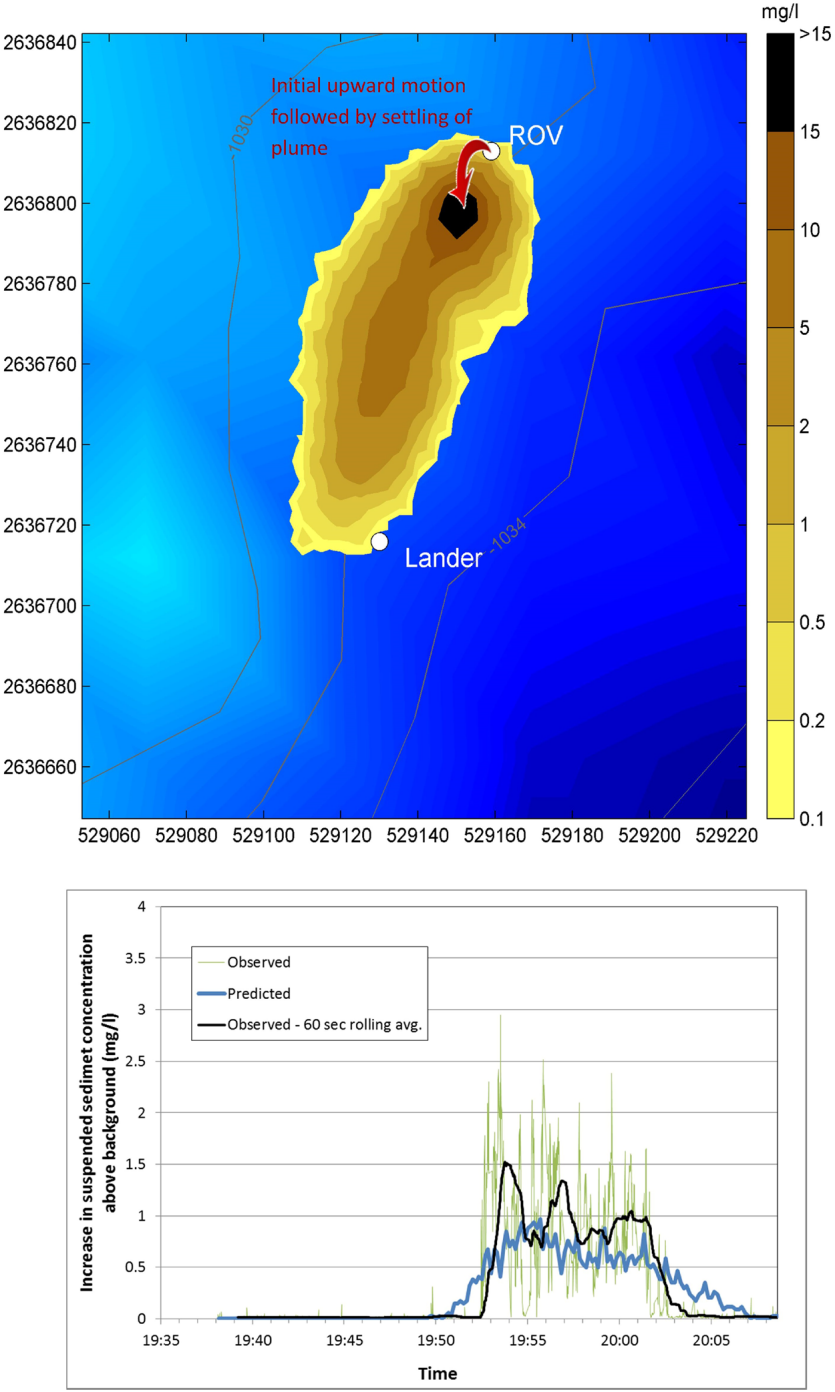
Top: Predicted increases in suspended sediment concentrations 1.5 m above the bed, 13 minutes into a plume experiment releasing 100 m from the Lander on the 18 November. East and west scale is in metres and contours are depth of the seafloor. Bottom: Comparison of observed and predicted sediment plumes at 1.5 m above the bed, same experiment.

The predicted current patterns were used to reproduce the *in situ* plume experiments. 13 out of the 19 successful plume experiments were modelled. Figure 6a shows an example of the predicted OBS suspended sediment concentrations 1.5 m above the bed during one of the experimental plumes, released 100 m distant from the Lander, on the 18 November. These results represent a snapshot of the model prediction of the plume 12 minutes after the start of plume release. The plume concentrations at this height are lower for the first 10 m or so from the ROV because of the initial vertical release of the plume followed by descent due to negative buoyancy and by particles settling. In Fig. 6b, the predicted suspended sediment concentration 1.5 m above the bed is compared with observations of the plumes concentrations during the same experiment. Additional model comparisons from the other 12 modelled experiments are presented in Supplementary Information Note 1.

The raw OBS signal varies over a wide range and this variation arises because of the difficulties of generating a constant rate of release of sediment using the ROV and the creation of “pulses” of sediment. The model, however, assumes a constant release rate and so predicts a much smoother variation. Nevertheless, when the model prediction is compared with a 60 second rolling time-average of the observations, it can be seen that the model compares favourably. Overall the model predicted the mean concentration measured at the lander with an accuracy (mean absolute error) of 51% (comparison presented in Supplementary Information Note 1).

During some of the plume experiments, the AUV was used to occupy and monitor a wide section through the water column at a range of 1 km down-stream of the lander. Results from the lander show both backscatter and ADCP signals in the water column whereas the AUV optical backscatter data showed no detectable plume at a range of 1000 m. The plume modelling corroborated this result, the predicted increases in plume concentration at a distance of 1 km being below 0.01 mg/l, i.e. less than the natural background variation.

### Numerical modelling of crust plumes with representative mining rates

**Figure 7:**
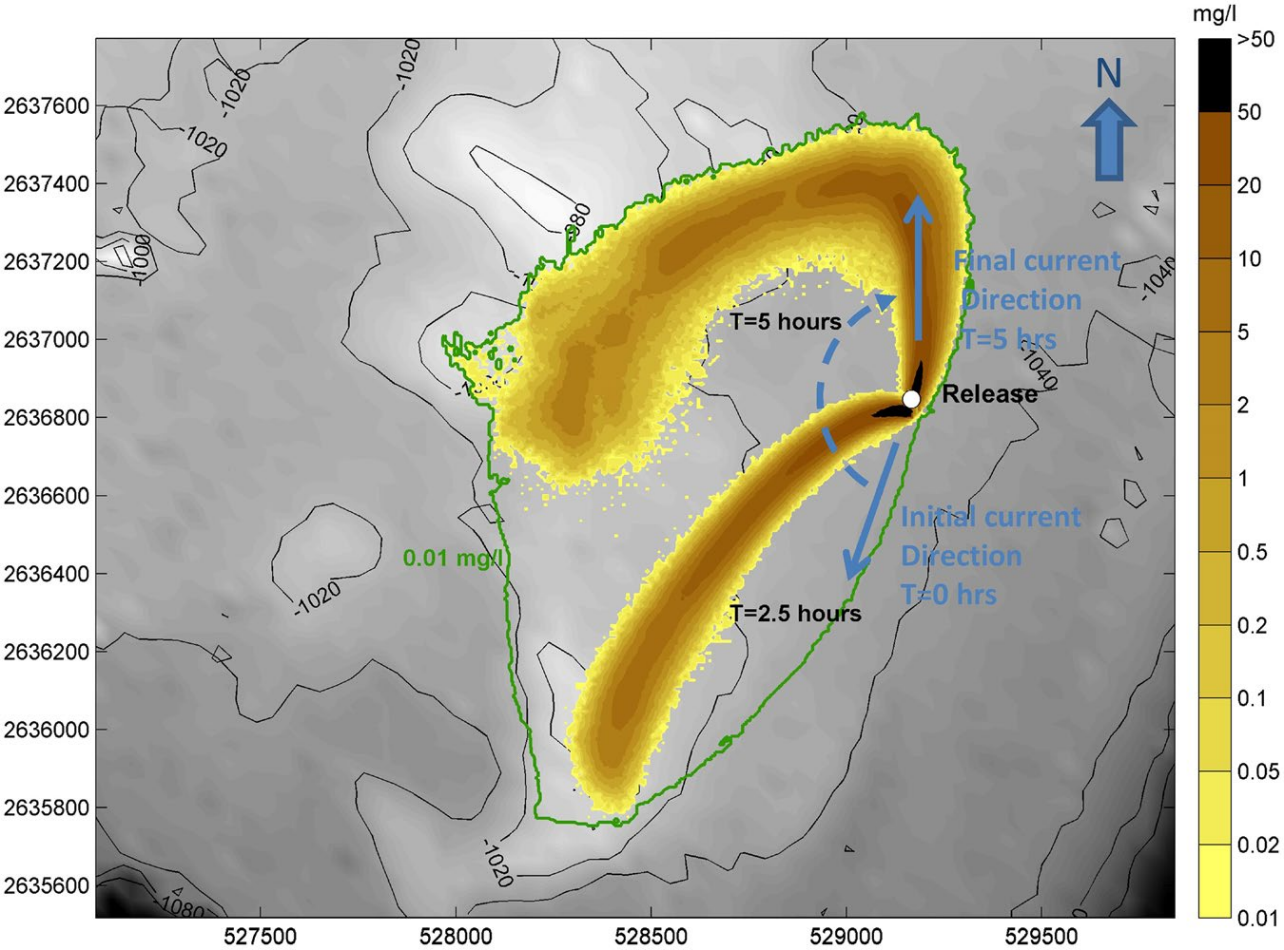
Snapshots of predicted increases in suspended sediment concentration (averaged over bottom 10 m of water column) resulting from 5 hour simulation with representative rate of release associated with mining. Snapshots shown at +2.5 hours and +5 hours. Envelope of increases greater than 0.01 mg/l over whole of simulation indicated by green line.

In this numerical experiment, the modelling for the sediment plume was repeated (with a stationary release) but over a longer duration using rates of release of fine crust particles (<63 µm) which are representative of the rates of release associated with mining of the crust (see methodology for the basis of the estimate). Figure 7 shows the results of a 5 hour simulation of continual release of fine sediment, at an average rate of 8.2 kg/s, with the distribution of settling velocities shown in Table 4. The figure shows snapshots of the plume (here at 2½ and 5 hours) and the envelope of predicted increases in suspended sediment concentration above 0.01 mg/l.

During the simulation, the current starts in a SSW direction and rotates clockwise, finishing in a north direction by the end of the simulation, in keeping with the observed tidal excursion for the summit of Tropic Seamount. The figure shows that the excursion of the plume towards the west and south (the principal direction of the currents during the simulation) is limited to around 1.4 km due to the combination of rotating tidal currents, flocculation and the limiting effect of background variation as discussed above.

Deposition during the numerical experiment was found to occur within 100 m of the release point. In reality, there would be additional deposition of some coarser crust aggregates which would settle out more rapidly. Because the release of these coarser particles takes place within a few metres of the bed, and the current speed is of the order of 0.1 m/s, and the settling velocity of these particles is of the order of cm/s, these coarser particles will settle within a few tens of metres from the source.

## Discussion

The studies described in this paper indicate that successful and detailed *in situ* measurements of sediment plumes can be undertaken in deep water, both in the near–field and the far-field. Whilst measurements of synthesised deep-sea mining plumes have been undertaken before, the present study level has for the first time combined novel methods of plume monitoring with detailed numerical modelling and laboratory measurements of settling velocity, to enable a plume dispersion model to be successfully validated to specific experiments. On this basis, and through considering the natural background suspended sediment variability, a more rigorous idea of the extent of plume dispersion arising from mining activities has been derived than in previous studies.

The modelling undertaken shows that the flow and sediment transport models, required for deep-sea plume dispersion, can provide confidence to regulators regarding the results of mining environmental assessments.

The experiments described have concentrated on providing model validation data for benthic plumes – a task which could in general be undertaken during the “collector test” phase of mining preparation as part of the environmental impact assessment (EIA) studies. Additionally, these techniques could equally be applied to mining plumes during full operations to confirm predictions made at the EIA stage and modify mining plans as required.

The measurements and modelling undertaken indicate that benthic plumes are limited by flocculation and by background turbidity signals and, in the case of seamounts as here, by rotational currents caused by internal tides. In our studies benthic plumes arising from disturbance by deep sea mining of cobalt-rich crusts were limited to within 1.4 km of the mining site and that deposition was limited to within 100 m of the disturbance. This result matches depositional evidence from surveys around *in situ* experiments simulating mining^8,20^. Note that flocculation also occurs in other deep sea mining environments^23,44^ and the natural variation in background sediment concentrations in some of these environments – the CCZ in particular - has been measured to be signification higher than that encountered in the present study (O(0.1) to 4 NTU^45,46^;). On this basis there is every reason to suppose that the footprint of plume impact – the area over which increases in plume concentration from mining are greater than the natural variation in turbidity – will be limited in similar ways to that found for the mining of crusts in this study.

One aspect not addressed in this study, and identified as a topic for further research, is the potential for the dispersion of fine sediment disturbed by mining to change over time as the mining continues. For the mining of crusts or massive sulphides, where a proportion of the mined rock will be lost to the water column and will and re-settle as fine material, there exists the possibility for fine sediment to increase in abundance over time in the vicinity of the mining - much as it can do with extended cutter suction dredging projects in shallow waters^47^. This build of fine sediment may lead to enhanced increases in turbidity as this fine material is re-worked. Mitigating against this possibility is the fact that this fine material is potentially viable ore (as recognised in the mining proposed at Solwara^4^) and mining companies will wish to design their systems to collect as much of this fine material as is possible, rather than lose it to the surroundings. This issue is less relevant for mining of nodules since the seabed substrate is silt/clay-sized and continued mining is unlikely to make this substrate any finer.

The results presented do not remove the environmental concerns associated with the mining of seamounts however they provide evidence that the dispersion of benthic plumes is likely to be far more localised than the footprint of mining effects hypothesised by some researchers (e.g. refs. ^16,22,48^). In consequence, benthic plumes arising from mining of deep-ocean seamounts (hosting a mineral resource on a flat summit) with Taylor caps are likely to be confined to the flat summit. The rotational nature of the currents and the absence of long dispersion distances suggests that interaction between ecology on the seamount slopes, or at distance from the seamount, is unlikely. In the case of the Tropic Seamount, extensive surveying by AUV has identified the presence of significant sponge and coral habitats on the summit^49,50^ which would be vulnerable to direct impact in any proposed mining areas and in the associated (and we assert on the basis of this study, limited) neighbouring zone(s) of dispersion and deposition. While further work is needed to assess the detailed significance of a full mining operation on the seamount ecology, we suggest a combination of plume monitoring and vulnerable species mapping can be used in future to adapt mining operations and minimise impact beyond the mine site.

## Methodology

### Field Measurements

Hydrodynamics at the site were studied using three fixed moorings at the location shown in Fig. 1 (measuring currents between 100 m and 3000 m depth and deployed for thirty days) together with ship-deployed ADCP and CTD casts. The moorings comprised an array of instrumentation to measure current velocities using Nortek Aquadopp DW Current Meters - on the northern and southern moorings - and an additional TRDI Workhorse Sentinel ADCP working at 300 kHz - on the summit mooring - and salinity and temperature using Seabird SBE37 CTDs moored at two separate heights above the seabed. Additional CTD casts (using the same instrumentation) were made at 30 locations as indicated in Fig. 1. These data were supported by sampling of the crusts, with video and photography of the seabed substrate and ecology. An autonomous underwater vehicle, Autosub6000, was used to extensively map the bathymetry over the seamount and acquire side-scan sonar imagery.

### Plume experiments

The plume experiments were undertaken at locations where the substrate was comprised of sediments and the bed showed little topographical variation. Key to the success of the plume experiments was: the siting of the lander; the positions of the sites at which the plumes were generated relative to the lander; and, due to the strong rotational tidal signature of the currents at the seamount crest, the experiment timing. Using the predicted time of high water and the near bed flow model predictions, experiments were timed to coincide with a period in the flow pattern where current directions were at their most consistent. Detailed experiment planning was undertaken to estimate, as best as possible, the times at which plumes should be generated. For each experiment the siting of the sediment plume generation with respect to the Lander was fine-tuned using a streamer to give the current direction as shown in Fig. 2a.

**Figure 8:**
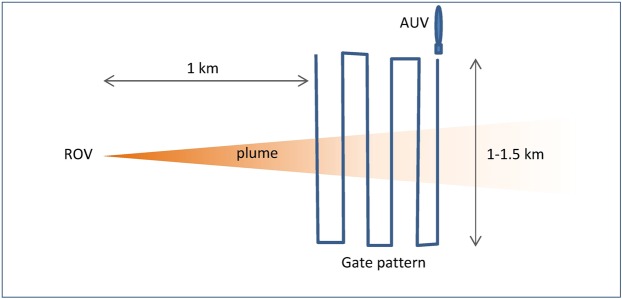
Deployment of the Autosub AUV during the plume experiments.

During the experiments, the ROV was used to generate the sediment plumes using a hydraulic pump mounted on the vehicle. The intake of the pump was directed into seabed sediments with the pump outlet mounted on the side of the ROV adjacent to one of the ROV’s down-thrusters, such that the output was directed upwards. Plumes were generated from locations, or sub-sites, at increasing distances from the lander with a view to characterising the sediments in the plume at different phases of its evolution. Plume experiments were conducted at three separate locations on the seamount crest (Fig. 1). Plume generation took place (over 10 minutes) with the ROV in fixed locations, as well as with the ROV moving along a transect, with sub-sites being located at distances of 25 m, 50 m and 100 m from the lander. 12 experiments were undertaken at each location (3 repeated experiments and one moving experiment, at each of the 3 sub-sites) giving 36 plume experiments in all, of which 19 experiments successfully measured a plume. Autosub6000 was flown through the predicted path of the plume at a distance of approximately 1 km down-stream for some of the experiments, occupying three depths (5 m, 20 m, and 25 m respectively) in a 2D gate-pattern (e.g. Fig. 8) 1–1.5 km wide for the entire duration of the experiments plus an hour, and used to measure turbidity within the plume at this distance.

**Figure 9:**
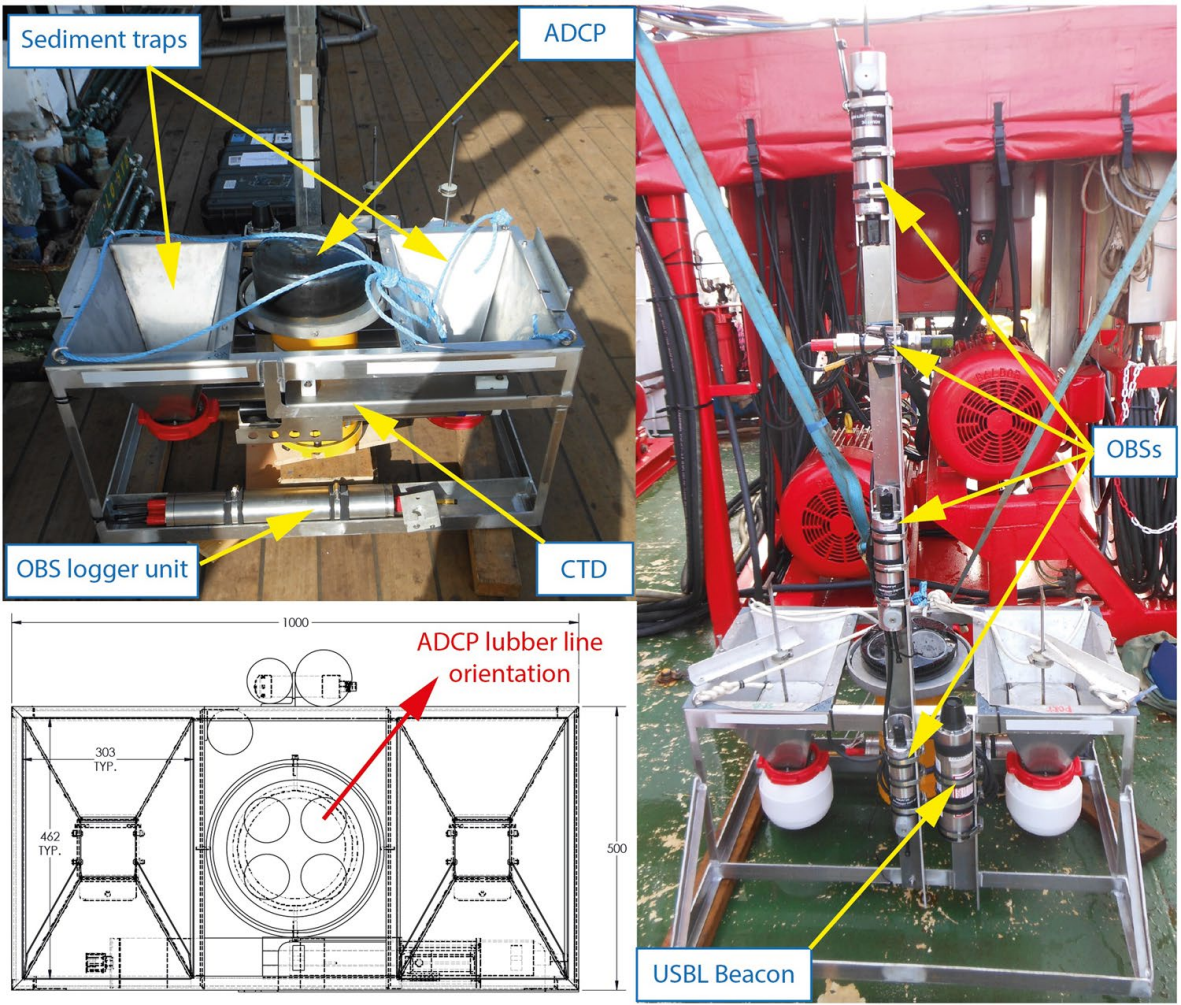
Annotated images of HR Wallingford’s benthic lander showing disposition of instrumentation. The red arrow indicates the lubber line of the ADCP’s internal compass.

The lander, shown in Fig. 9, was constructed from marine grade aluminium and measured 1.0 × 0.5 × 0.3 m with a 1.2 m high mast. The platform facilitated the deployment of a TRDI 600 kHz Workhorse Sentinel ADCP, a Seabird SBE37 CTD sensor, three Aquatec 210TY optical backscatter sensors (OBSs) positioned at heights of 0.5 m, 1.0 m and 1.5 m above the bed and a further Valeport OBS (configured at much higher concentrations) at 1.3 m above the bed. The lander was also equipped with two funnel-shaped sediment traps which could be opened once the lander was on the seabed and closed before its recovery.

The OBS data was recorded from the benthic lander during the plume experiments to provide suspended sediment concentration (SSC) data within the plumes. However, a calibration is necessary to convert from raw measurements in formazin turbidity units (FTU) to SSC in mg/l. Instruments used during the cruise were calibrated using a laboratory dilution method. Sediment samples collected during each experiment were added to seawater from the seamount site to make up a suspension of known SSC. The optical sensors were then “dipped” into this known concentration and their FTU readings recorded, with the process being repeated over a range of SSC. The calibration was undertaken for each OBS instrument for each of the three sets of plume experiments.

### Laboratory settling tests

During the cruise samples of the sediment and crust and (near-bed) sea water were taken and stored in refrigerated conditions for later use in the laboratory settling experiments. These experiments created 20 mg/l and 100 mg/l samples sea water and sediment/crust debris and measured the settling velocities of the floc populations in these 1 litre samples using the LabSFLOC video-imaging technique^26,33^. This utilises a high magnification 2.0MP Grasshopper monochrome digital video camera fitted with a Sill TZM 1560 Telecentric, 0.66 (1:1.5) magnification, F4, macro lens located behind a 5 mm thick glass faceplate (this type of lens (provided by Stemmer Imaging Ltd) has the advantage of minimising any pixel distortion - maximum distortion of 0.6%), to observe particles settling in a Perspex settling column allowing for minimal disruption of the particles. The video camera, positioned nominally 75 mm above the base of the column, views all particles in the centre of the column that pass within a 1 mm depth of field, 45 mm from the lens. The video camera utilizes a back-illumination system whereby floc images are viewed as silhouettes i.e. particles appear dark on a light background. This reduces image smearing, and renders the floc size and structure more visible. This back-illumination is provided by a CCS LDL-TP-43/35-BL, 43 ×35 mm, homogeneous blue (470 nm) LED backlit panel (also provided by Stemmer Imaging Ltd) located at the rear of the settling column.

As part of the measurement protocol the proportion of the total mass of the flocs which settled slowly and were too small (<40 microns) to be reliably detected by the video camera was also assessed by additional settling tests. These tests involved the measurement of the decay in suspended sediment concentration of 100 mg/l samples of sediment and crust debris over a 3 hour period. The decay in concentration over time was measured in 1 litre glass jars by taking small samples of the solution by pipette, the sediment/crust mass being determined by filtration and weighing.

### Numerical modelling

The flow model used for this study was TELEMAC-3D^51^, which solves the full Navier-Stokes equations and the transport-diffusion equations of intrinsic quantities (temperature, salinity, concentration). It uses a fully unstructured mesh that makes it particularly useful for creating suitable meshes with horizontal layers in the open ocean (usually most useful for capturing saline and thermal gradients), as well as adapting vertical layers to steep bathymetric features (e.g. on coasts, or as here, on the flanks of a seamount). It is employed in this study as a local model (scale: 25,000 km^2^) in combination with a regional ocean model, the global Mercator model^52^, the latter providing the required boundary conditions to the local-scale. The TELEMAC-3D model was run with a resolution varying from 150 m at the seamount in the vicinity of the plume experiments to 5 km at the model boundaries. The model had 49 model planes and, on the summit of the seamount the lowest of these layers are distributed at 0, 1, 2, 5, 10, 15, 20, 25, 30, 35, 40, 50, 55 metres above the bed. To represent turbulence the model used a mixing- length approach^53^ with damping of turbulence described by Viollet^54^.

Boundary conditions of tidal level and tidal current were taken from the TPXO model^55^ assuming a uniform barotropic current through the vertical. Non-tidal ocean current boundary conditions of water level and current were taken from the global Mercator Ocean circulation model^40^. The water levels and currents from these two sources were combined and, in order to reproduce accurately the water level including the tide, the water level boundary condition was imposed without relaxation. The total (depth varying) current, salinity and temperature on the boundary were imposed using a sponge layer approach. The initial conditions of salinity and temperature were taken from the average vertical water column CTD profiles taken during the cruise. Wind forcing was applied using winds predicted by the ERA Interim, Daily forecast (https://www.ecmwf.int).

Modelling of the plume experiments was reproduced using the SEDPLUME-RW 3D lagrangian dispersion model^56^ which incorporates a near-field integral plume model^57,58^ (based on the formulation by Lee and Cheung^59^) to reproduce the rapid initial mixing caused by the plume jet following release. In the far-field phase turbulent mixing is reproduced through randomly moving particles by small amounts based on the eddy diffusivity, although the main mixing effect is the large-scale shear dispersion caused by spatial gradients in current speed^60^. The SEDPLUME-RW model used the TELEMAC-3D predictions of current to advect the plume, predicting mean concentration increases over the ranges: 0–1 m, 1–2 m, 2–5 m, 5–10 m, 10–15 m, 20–25 m, 25–30 m, 35–40 m, 40–45 m, 45–50 m and 50–55 m above the bed (these layers corresponding to the intervals between the TELEMAC-3D layers). The sediment plume simulations were run on a 200 m by 200 m grid with 2.5 m resolution and a 10 second time step using approx. 150,000 particles. The near-field sub-model was run with a 0.01 s time step and was used to calculate the mixing over the first 20 seconds of plume release on each time-step of the far-field model. The crust mining simulation was undertaken on a 2.3 km × 2.8 km grid with 10 m resolution, using approx. 5,200,000 particles and a 10 second time step, without the near-field mixing sub-model. The SEDPLUME-RW model represented turbulence using mixing length approach^61^ with Munk-Andersen turbulence damping^62^.

### Estimate of release rates of sediment for the numerical simulations

The estimate of source terms for the sediment plume tests was deduced by estimating the volume of sediment removed from the bed on each plume experiment using multi-beam and photography and assuming a dry density of the seabed associated with loose sand (approx. 1500 kg/m^3^ ^35^). The estimate of the fine sediment source term for the mining release was estimated using (a) an assumption of mining of 2 M tonnes of resource per year; (b) an assumed efficiency of 70% in the mining process; (c) the geotechnical properties of crust are similar to those of a weak weathered rock (e.g. ref. ^63^); (d) an assumption that around 30% of the mined crust would break up into particles smaller than 63 µm, analogously to weathered weak rock being dredged by cutter suction dredger (based on published and non-published results from the dredging industry, e.g. refs. ^64,65^); and, (e) 30% of the crust dredged is not collected by the suction pipe^66–68^, by analogy with cutter suction dredging of weak rock, and the fine sediment component of this sediment is resuspended. These assumptions result in an estimated representative release rate for fine crust particles (diameter <63 µm) of 8.2 kg/s.

## Supplementary information

Supplementary Information.

LP1_SSC_Plume_Data.

LP2_SSC_Plume_Data.

LP3_SSC_Plume_Data.

## Data Availability

*Model code:* The TELEMAC3D flow model code is open source and can be downloaded from the website at http://www.opentelemac.org. All files used to run the flow model for the periods used in the study are described in Supplementary Information Note 2 and are available from the BODC Published Data Library: 10.5285/9c949855-dbd5-6e9e-e053-6c86abc0f145. The dispersion model SEDPLUME-RW used in the study is a commercial code developed by HR Wallingford and the code for this model is not provided. However, a complete description of the model formulation is provided in Supplementary Information Note 3 together with full information regarding the input parameters for each of the dispersion model runs. *Data:* The observational data for each of the validation comparisons (those shown in the main text and in Supplementary Information Note 1) are provided in the Supplementary Information. Additional raw and processed data associated with this study can be directly downloaded from the BODC Published Data Library: 10.5285/8df4ff45-ca9c-040f-e053-6c86abc07386.
