# Supplementary material for: Measurement and modelling of deep sea sediment plumes and implications for deep sea mining
Source: Sci Rep. 2020 Mar 19;10:5075. doi: 10.1038/s41598-020-61837-y (PMC7081334; doi:10.1038/s41598-020-61837-y)
Supplement: Supplementary file 1 — Supplementary Information. [file 41598_2020_61837_MOESM1_ESM.docx]

**Measurement and modelling of deep sea sediment plumes and implications for deep sea mining**

Jeremy Spearman, Jon Taylor, Neil Crossouard, Alan Cooper, Michael Turnbull, Andrew Manning, Mark Lee & Bramley Murton

**Supplementary Information part 1**

**Further modelling outputs**

**Contents**

Supplementary Figure S1-1

Supplementary Figure S1 -2

Supplementary Figure S1-3

Supplementary Figure S1-4

Supplementary Figure S1-5

Supplementary Figure S1-6

Supplementary Figure S1-7

Supplementary Figure S1-8

Supplementary Figure S1-9

Supplementary Figure S1-10

Supplementary Figure S1-11

Supplementary Figure S1-12

Supplementary Figure S1-13

Supplementary Figure S1-14


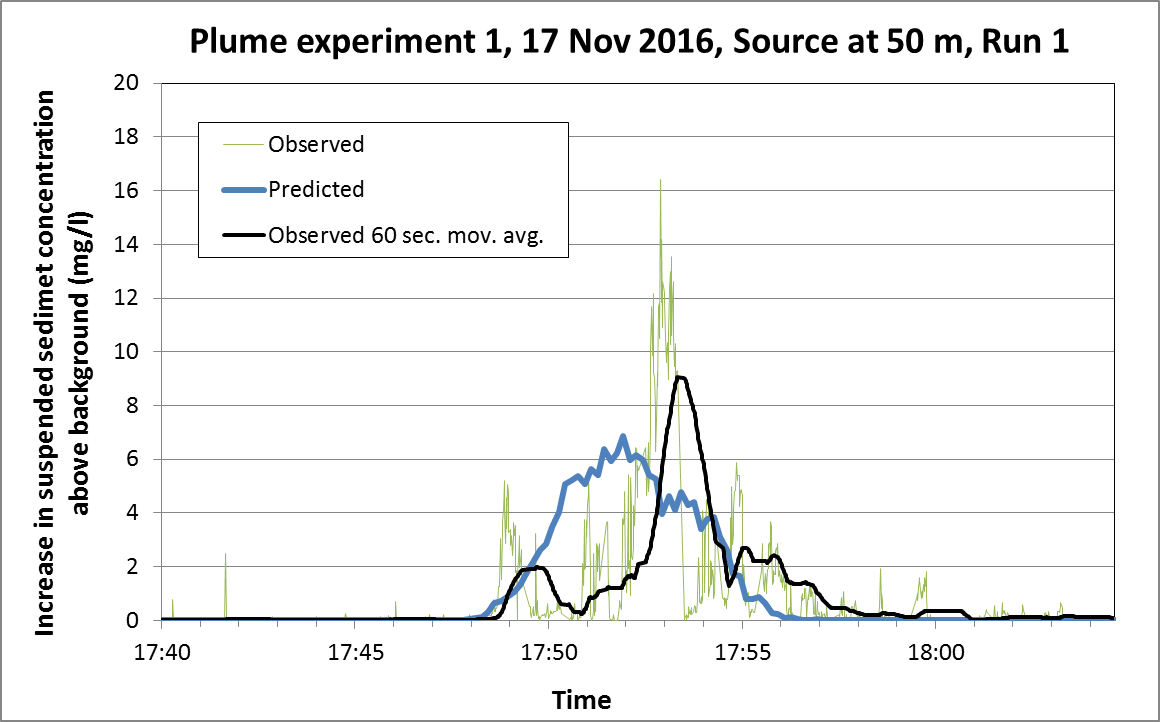


**Supplementary Figure S1-1** Comparison of predicted and observed suspended sediment concentrations at Lander 1.5 m above the bed, Plume experiment 1, 17 November 2016, Source at 50 metres, Run 1


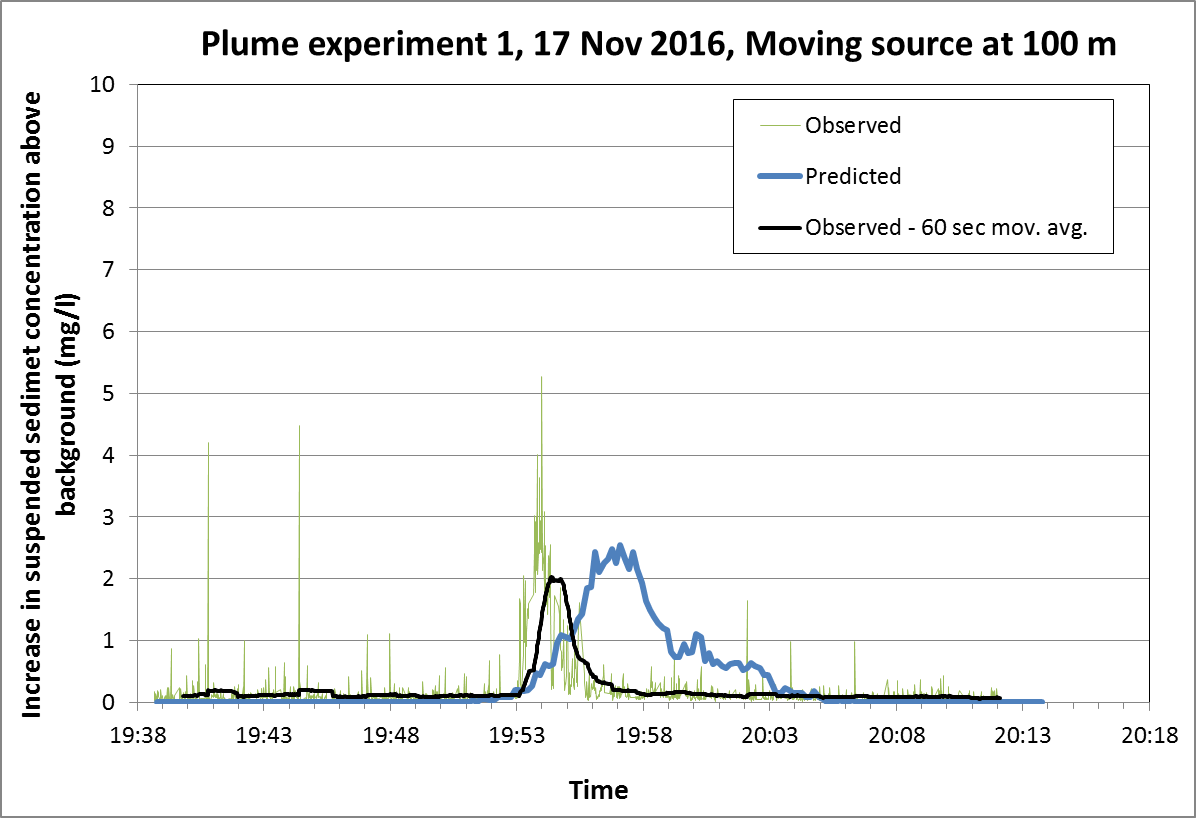


**Supplementary Figure S1-2** Comparison of predicted and observed suspended sediment concentrations at Lander 1.5 m above the bed, Plume experiment 1, 17 November 2016, Moving source at 100 metres


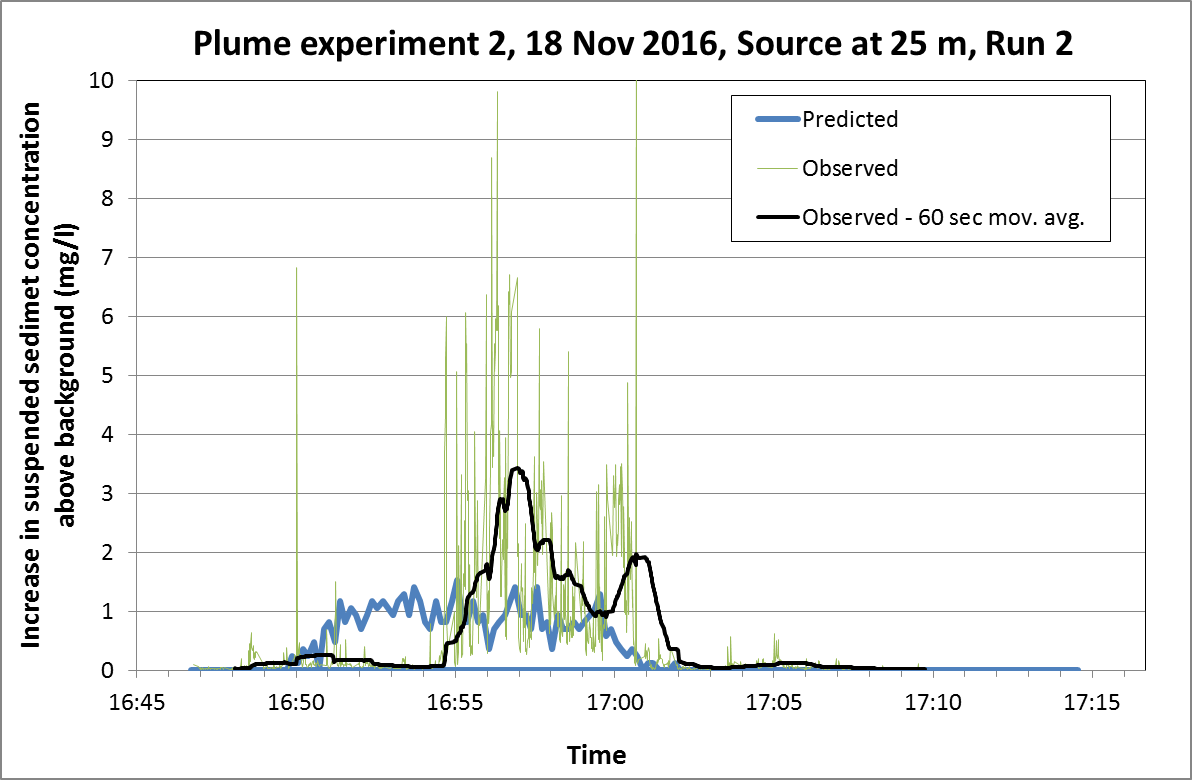


**Supplementary Figure S1-3** Comparison of predicted and observed suspended sediment concentrations at Lander 1.5 m above the bed, Plume experiment 2, 18 November 2016, Source at 25 metres, Run 2


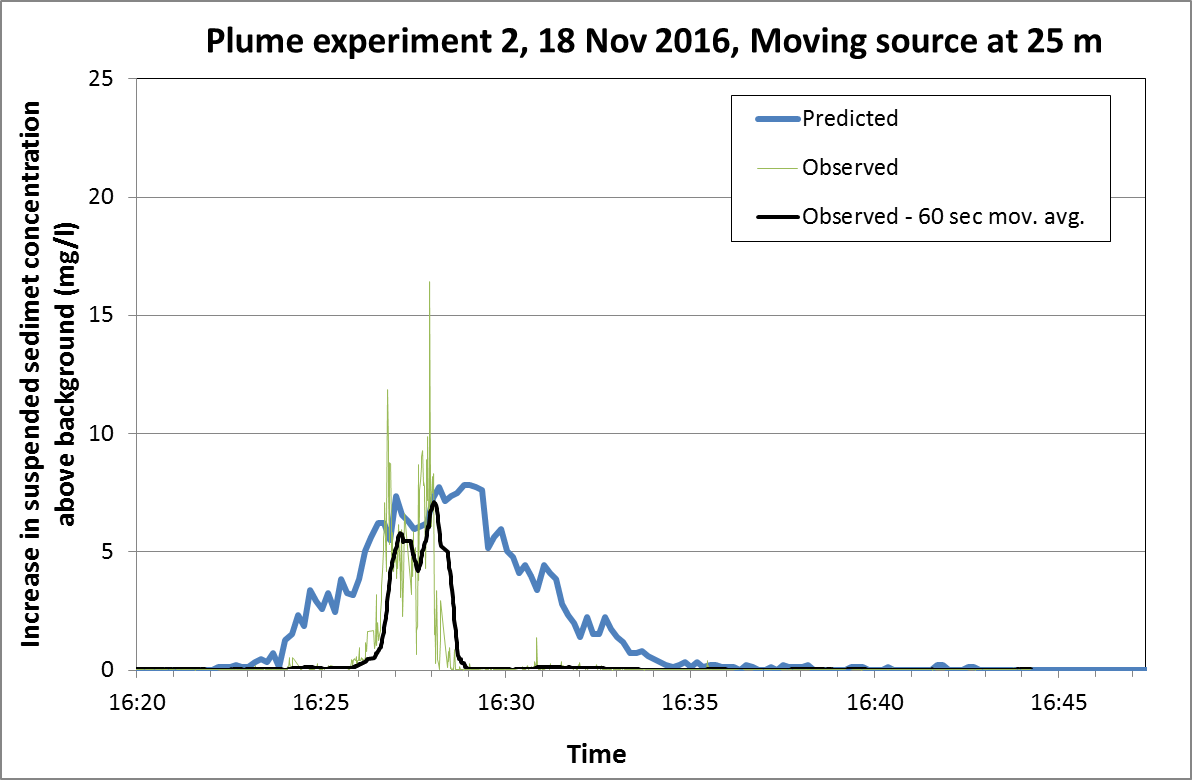


**Supplementary Figure S1-4** Comparison of predicted and observed suspended sediment concentrations at Lander 1.5 m above the bed, Plume experiment 2, 18 November 2016, Moving Source at 25 metres


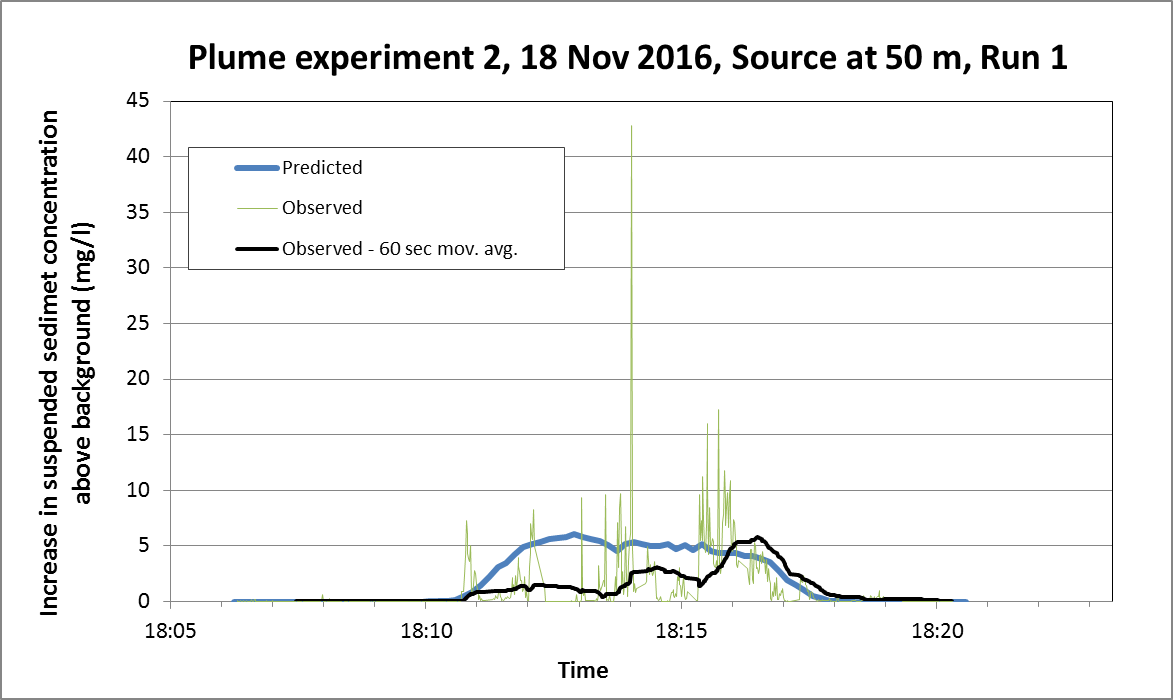


**Supplementary Figure S1-5** Comparison of predicted and observed suspended sediment concentrations at Lander 1.5 m above the bed, Plume experiment 2, 18 November 2016, Source at 50 metres, Run 1


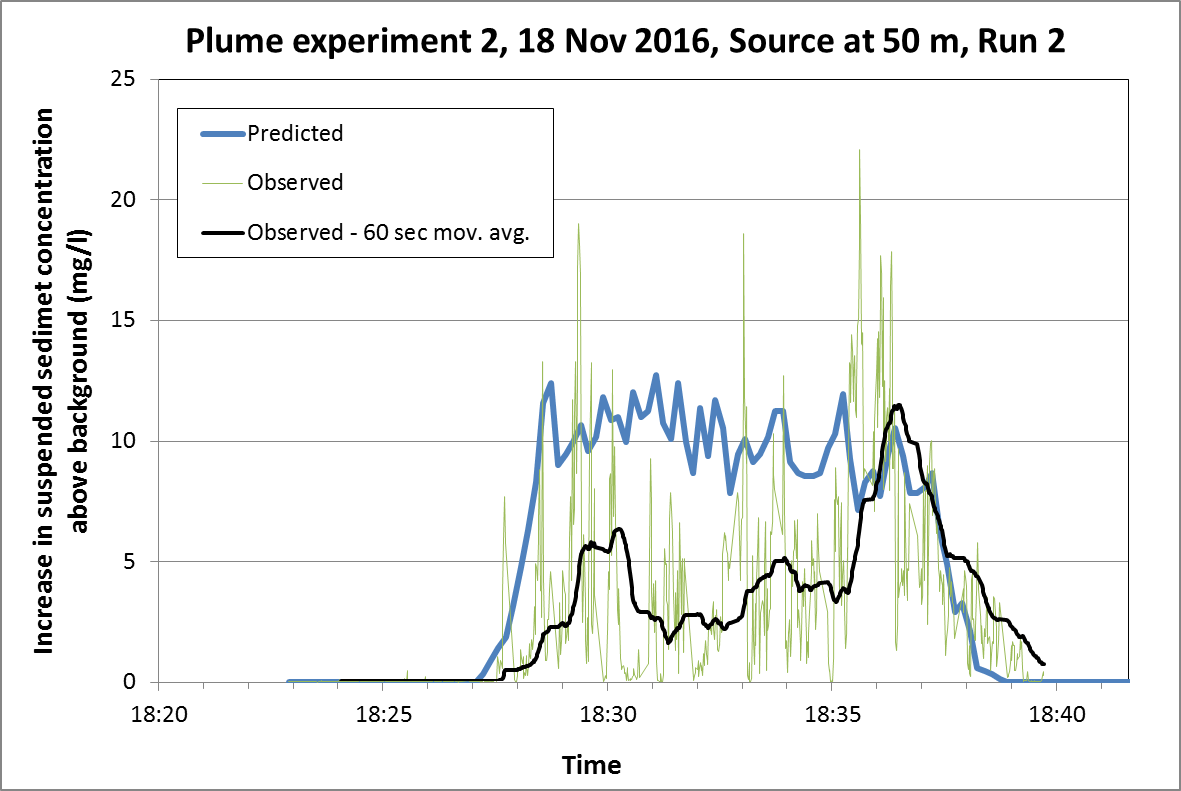


**Supplementary Figure S1-6** Comparison of predicted and observed suspended sediment concentrations at Lander 1.5 m above the bed, Plume experiment 2, 18 November 2016, Source at 50 metres, Run 2


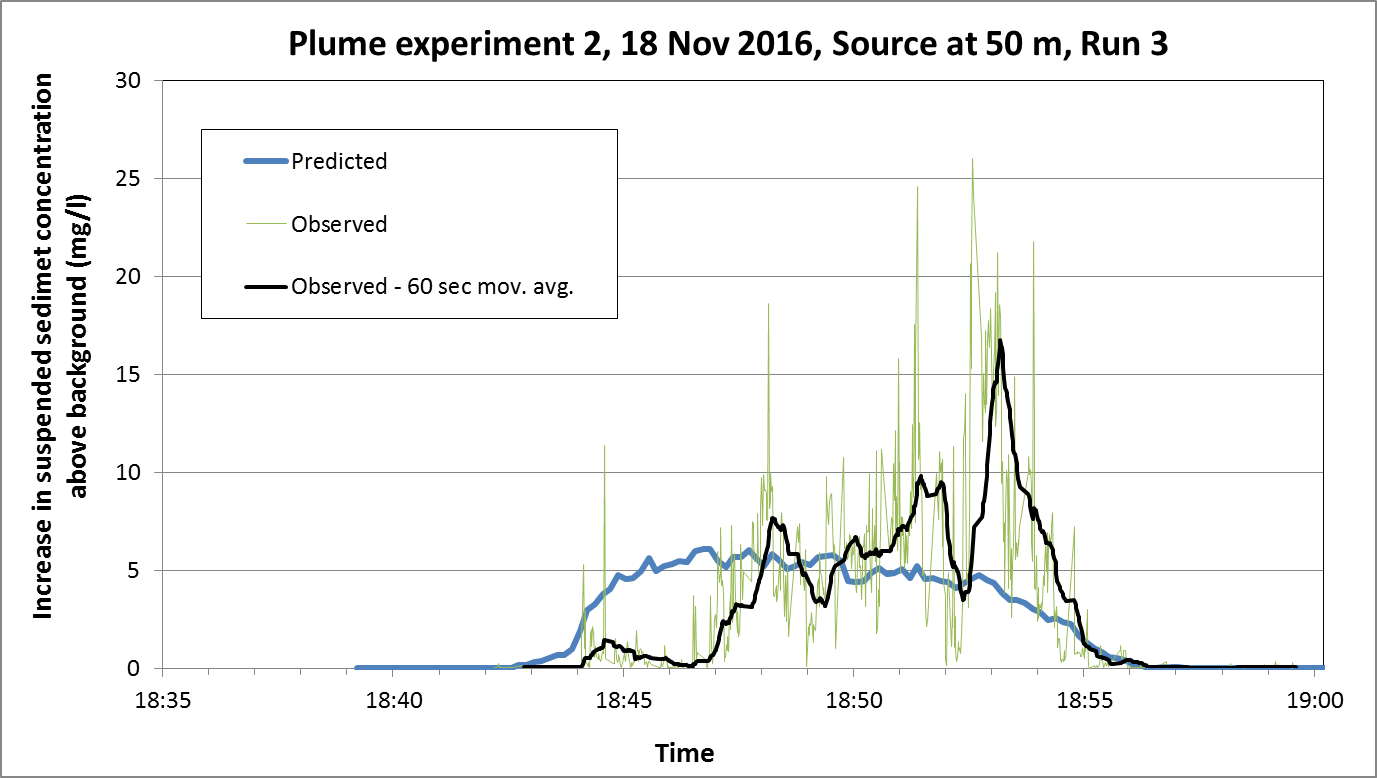


**Supplementary Figure S1-7** Comparison of predicted and observed suspended sediment concentrations at Lander 1.5 m above the bed, Plume experiment 2, 18 November 2016, Source at 50 metres, Run 3


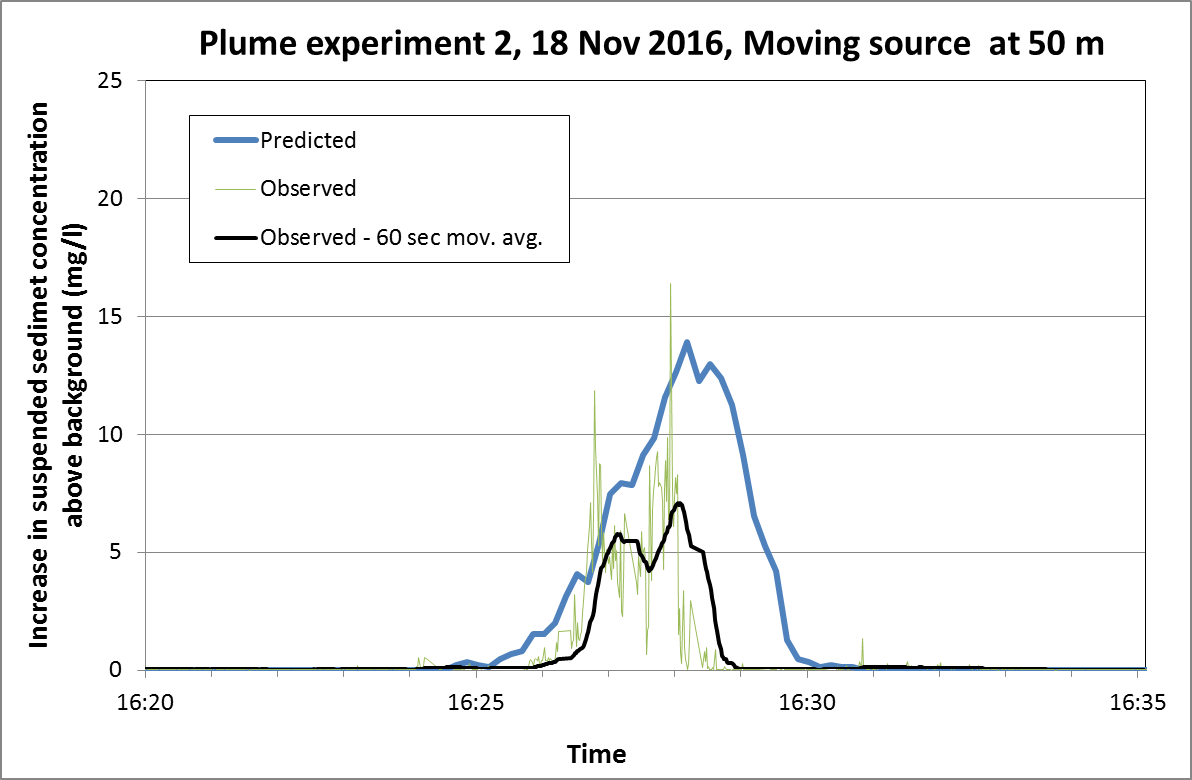


**Supplementary Figure S1-8** Comparison of predicted and observed suspended sediment concentrations at Lander 1.5 m above the bed, Plume experiment 2, 18 November 2016, Moving source at 50 metres


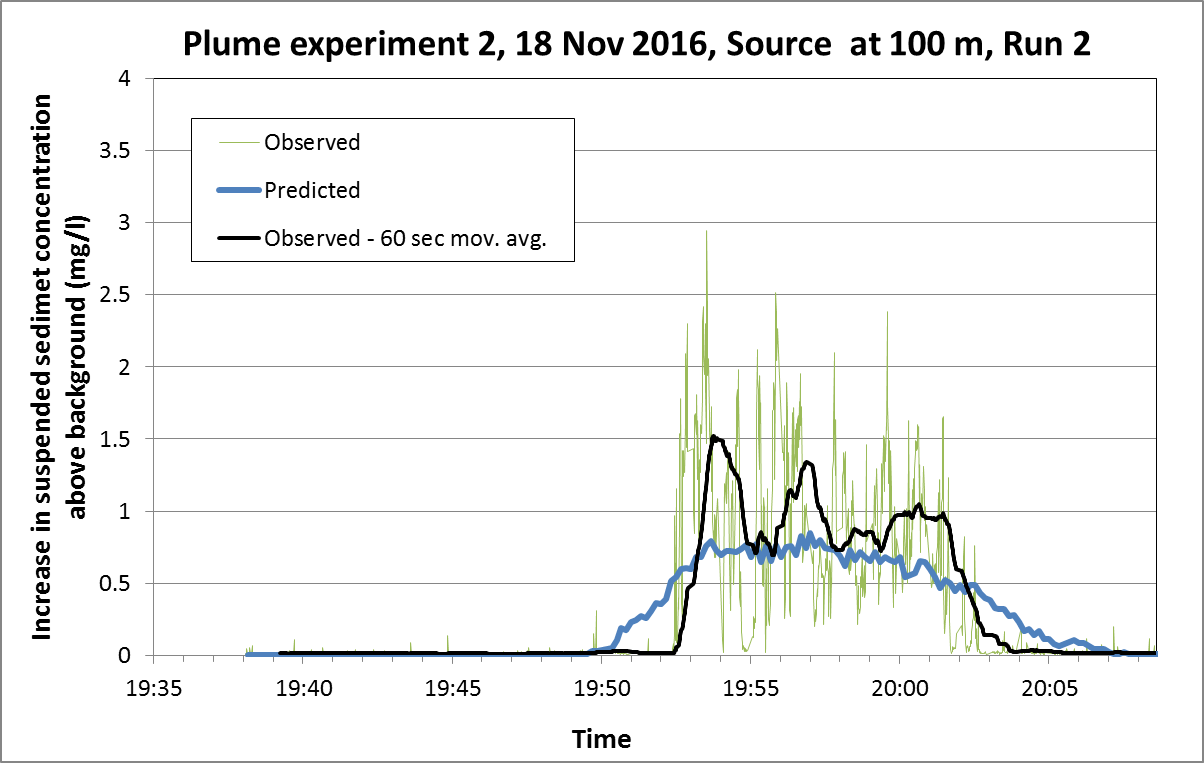


**Supplementary Figure S1-9** Comparison of predicted and observed suspended sediment concentrations at Lander 1.5 m above the bed, Plume experiment 2, 18 November 2016, Source at 100 metres, Run 2


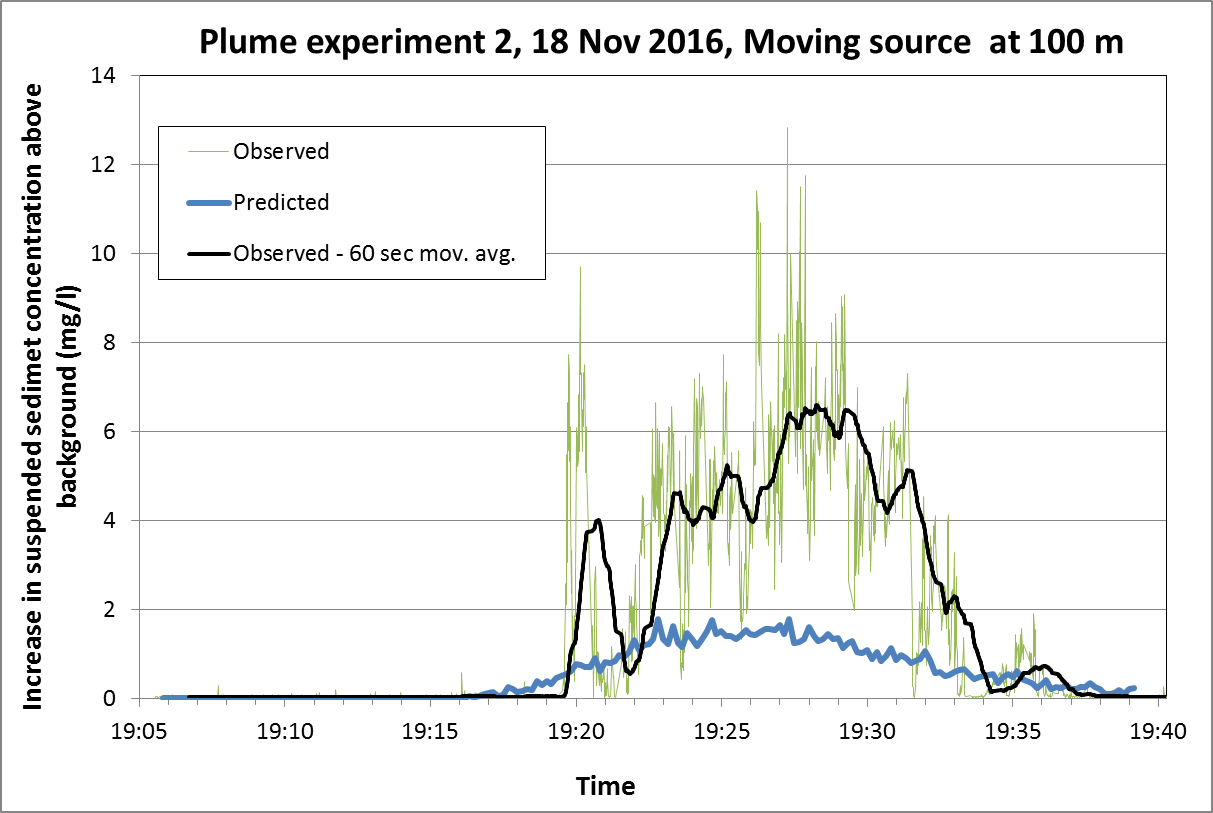


**Supplementary Figure S1-10** Comparison of predicted and observed suspended sediment concentrations at Lander 1.5 m above the bed, Plume experiment 2, 18 November 2016, Moving source at 100 metres


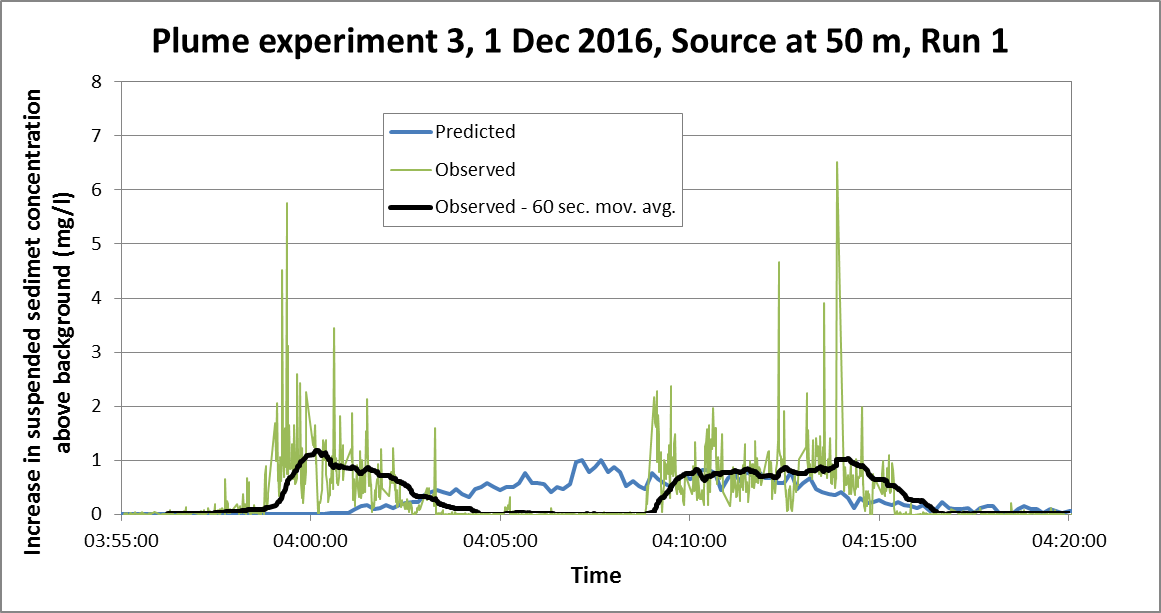


**Supplementary Figure S1-11** Comparison of predicted and observed suspended sediment concentrations at Lander 1.5 m above the bed, Plume experiment 3, 1 December 2016, Source at 50 metres, Run 1


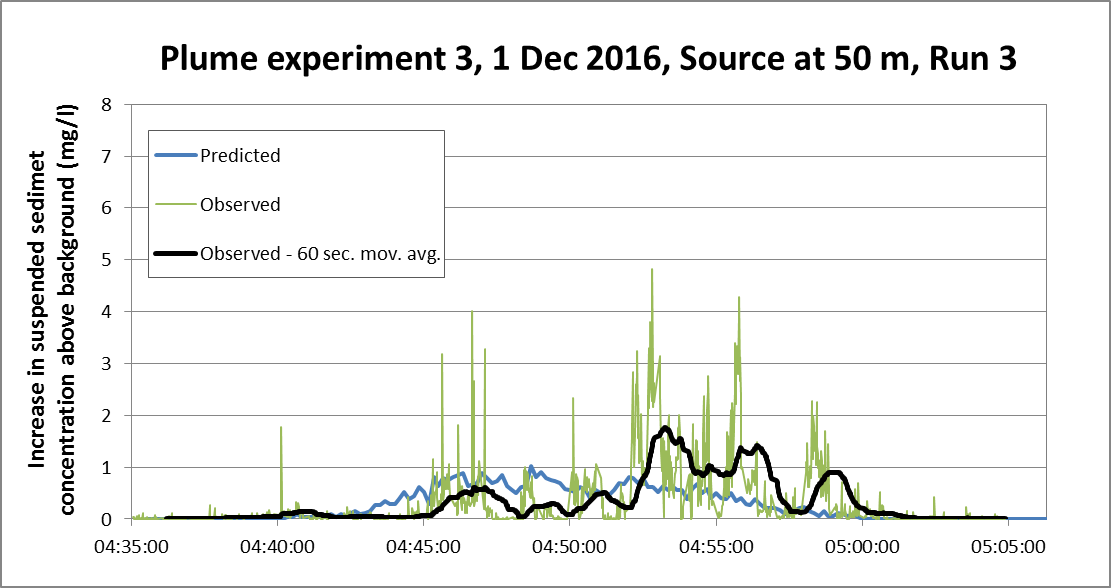


**Supplementary Figure S1-12** Comparison of predicted and observed suspended sediment concentrations at Lander 1.5 m above the bed, Plume experiment 3, 1 December 2016, Source at 50 metres, Run 3


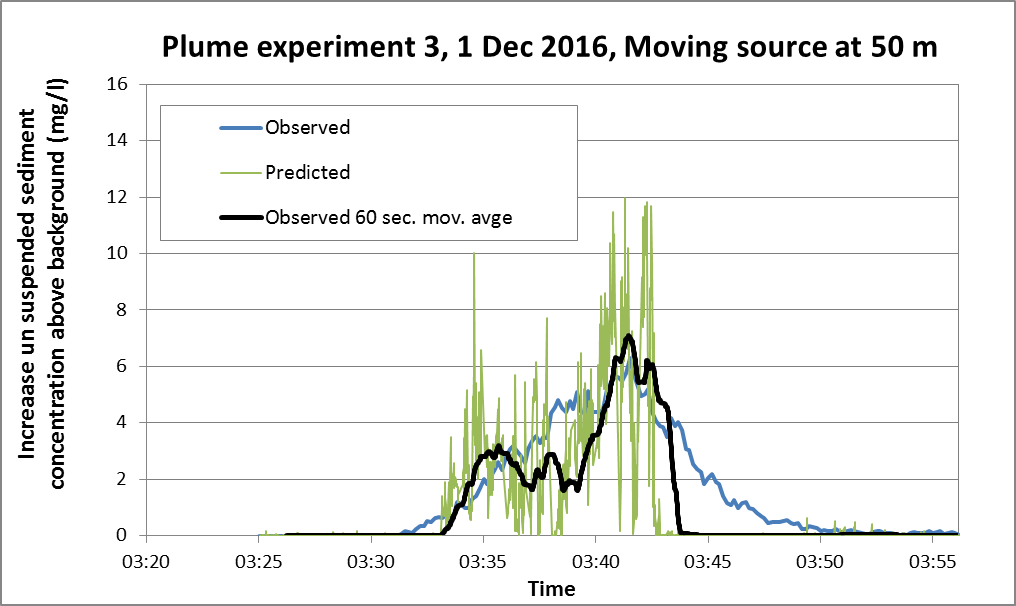


**Supplementary Figure S1-13** Comparison of predicted and observed suspended sediment concentrations at Lander 1.5 m above the bed, Plume experiment 3, 1 December 2016, Moving source at 50 metres, Run 3


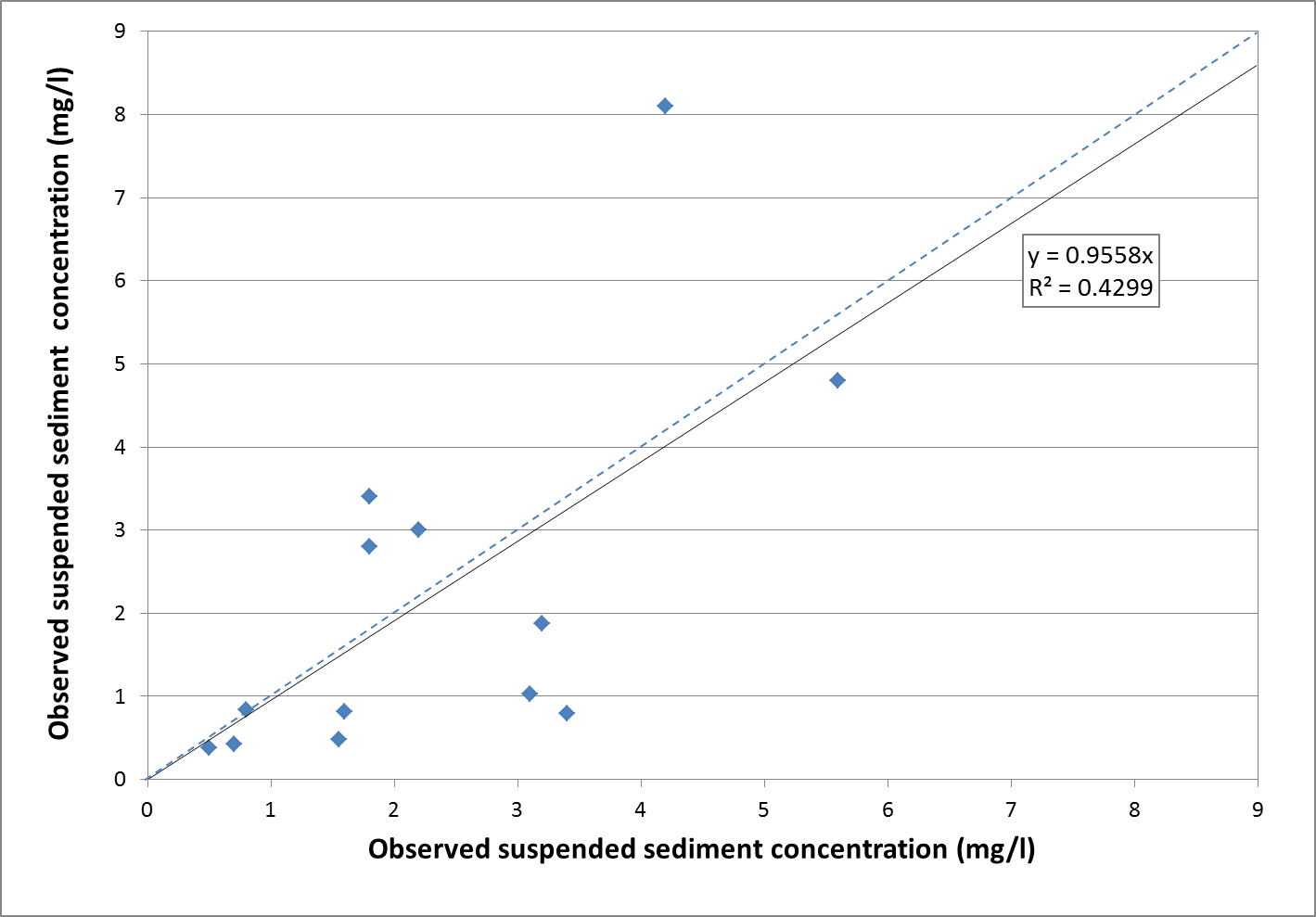


**Supplementary Figure S1-14** Comparison of predicted and observed suspended sediment concentrations, all modelled experiments. Dotted line: line of perfect fit. Continuous line: best linear fit through data points.

**Measurement and modelling of deep sea sediment plumes and implications for deep sea mining**

Jeremy Spearman, Jon Taylor, Neil Crossouard, Alan Cooper, Michael Turnbull, Andrew Manning, Mark Lee & Bramley Murton

**Supplementary Information part 2**

**Modelling information**

**Contents**

- 1. Description of flow modelling for experiments on 17 and 18 November
  2. Description of flow modelling for experiments on 1 December
  3. Description of plume modelling for experiments on 17 and 18 November
  4. Description of plume modelling for experiments on 1 December
  5. Description of longer plume experiment simulation
  6. Description of flow and plume modelling for simulation of mining
  7. **Description of flow modelling for experiments on 17 and 18 November**

The flow modelling for the experiments of **17 and 18 November** was undertaken using the open source TELEMAC software ([*http://www.opentelemac.org*](http://www.opentelemac.org)) using version 6.3 release 2. The flow model run lasted for 7 days starting at 00:00am 14 November. The files used, together with the boundary condition files (using outputs from the global TPXO tidal model and the MERCATOR ocean model) are listed below. The files themselves are BODC Data Library:

https://doi.org/10.5285/9c949855-dbd5-6e9e-e053-6c86abc0f145.

STEERING FILE : cas3d_seamount_255_v6p3r2

FORTRAN FILE : princi3d_seamount_254_v6p3r2.f

GEOMETRY FILE : geom_seamount_13_sp.slf

BOUNDARY CONDITIONS FILE : geom_seamount_13_2.cli

BINARY DATA FILE 1 : phys124_14nov2016_C_spg2_g13.slf

(This file contains data downloaded from Mercator Ocean for the period 14 Nov 2016 to 20 Nov 2016 and is used to provide non-tidal boundary conditions to the TELEMAC model. Python routines were used to read the Mercator Ocean output and to interpolate this output onto the TELEMAC mesh. The time in this file is relative to 14 November 2016).

PREVIOUS COMPUTATION FILE : phys124_14nov2016_C_ini_g13.slf

WIND CONDITIONS FILE : wind01_01nov16_jc142.txt

SALINITY CONDITIONS FILE : initial_temp_sal_01.txt

TPXO WATER LEVEL FILE : h_tpxo7.2

TPXO CURRENTS FILE : u_tpxo7.2

- 1. **Description of flow modelling for experiments on 1 December**

The flow modelling for the experiments of **1 December** was undertaken using the open source TELEMAC software ([*http://www.opentelemac.org*](http://www.opentelemac.org)). The flow model run lasted for 9 days starting on 00:00am 27 November. The files used, together with the boundary condition files (using outputs from the global TPXO tidal model and the MERCATOR ocean model) are listed below. The files themselves are BODC Data Library:

https://doi.org/10.5285/9c949855-dbd5-6e9e-e053-6c86abc0f145.

STEERING FILE : cas3d_seamount_254_v6p3r2

FORTRAN FILE : princi3d_seamount_254_v6p3r2.f

GEOMETRY FILE : geom_seamount_13_sp.slf

BOUNDARY CONDITIONS FILE : geom_seamount_13_2.cli

BINARY DATA FILE 1 : phys124_27nov2016_E_spg2_g13.slf

(This file contains data downloaded from Mercator Ocean for the period 27 Nov 2016 to 7 Dec 2016 and is used to provide non-tidal boundary conditions to the TELEMAC model. Python routines were used to read the Mercator Ocean output and to interpolate this output onto the TELEMAC mesh. The time in this file is relative to 27 November 2016).

PREVIOUS COMPUTATION FILE : phys124_27nov2016_E_ini_g13.slf

WIND CONDITIONS FILE : wind01_27nov16_jc142.txt

SALINITY CONDITIONS Fi9ILE : initial_temp_sal_01.txt

TPXO WATER LEVEL FILE : h_tpxo7.2

TPXO CURRENTS FILE : u_tpxo7.2

- 1. **Description of plume modelling for experiments on 17 and 18 November**

The plume modelling for the experiments of **17 and 18 November** was undertaken using the lagrangian 3D plume dispersion SEDPLUME –RW (developed by HR Wallingford). This model was used to undertake simulations of 35 to 50 minutes (as required for the plume to pass by the lander). The model used the hydrodynamic input from the TELEMAC run starting on 14 November and was run for the following experiments (Table S2-1):

Table S2-1 Description of modelled plume experiments for 17 and 18 November

| **Day** | **Plume Experiment** | **Experiment run** | **Time of start of experiment** | **Modelled period of release** |
| --- | --- | --- | --- | --- |
| 17 | 1 | 50 m from Lander, Run 1 | 17:35 | 10 mins |
| 17 | 1 | 100 m from Lander, Moving source | 19:39 | 9 mins |
| 18 | 2 | 25 m from Lander, Run 2 | 16:47 | 10 mins |
| 18 | 2 | 25 m from Lander, Moving source | 16:19 | 8 mins |
| 18 | 2 | 50 m from Lander, Run 1 | 18:07 | 6 mins |
| 18 | 2 | 50 m from Lander, Run 2 | 18:23 | 10 mins |
| 18 | 2 | 50 m from Lander, Run 3 | 18:39:30 | 6 mins |
| 18 | 2 | 50 m from Lander, Moving source | 17:54 | 5 mins |
| 18 | 2 | 100 m from Lander, Run 2 | 19:39 | 10 mins |
| 18 | 2 | 100 m from Lander, Moving sources | 19:06 | 15 mins |

The locations of the Lander and ROV source during these experiments are presented in Table S2-2.

Table S2-2 Locations of ROV source and Lander measurements during modelled plume experiments for 17 and 18 November

| **Day** | **Plume Experiment** | **Experiment run** | **Position (UTM 27)** | | | |
| --- | --- | --- | --- | --- | --- | --- |
|  |  |  | **ROV** | | **Lander** | |
|  |  |  | **E (m)** | **N (m)** | **E (m)** | **N (m)** |
| 17 | 1 | 50 m from Lander, Run 1 | 530336 | 2640660 | 530342 | 2640615 |
| 17 | 1 | 100 m from Lander, Moving source   - Start - End | 530415  530441 | 2640690  2640643 |  |  |
| 18 | 2 | 25 m from Lander, Run 2 | 529112 | 2636733 | 529130 | 2636716 |
| 18 | 2 | 25 m from Lander, Moving source   - Start - End | 529124  529113 | 2636740  2636733 |  |  |
| 18 | 2 | 50 m from Lander, Run 1 | 529124 | 2636764 |  |  |
| 18 | 2 | 50 m from Lander, Run 2 | 529130 | 2636764 |  |  |
| 18 | 2 | 50 m from Lander, Run 3 | 529133 | 2636765 |  |  |
| 18 | 2 | 50 m from Lander, Moving source   - Start - End | 529108  529131 | 2636764  2636765 |  |  |
| 18 | 2 | 100 m from Lander, Run 2 | 529159 | 2636813 |  |  |
| 18 | 2 | 100 m from Lander, Moving sources   - Start - End | 529126  529170 | 2636819  2636811 |  |  |

The model parameters used in the plume model run are shown in Table S2-3 and the release rates used are shown in Table S2-4. Comparisons of the predicted and observed suspended sediment concentrations at the Lander are shown in Figures S1-1 to S1-10 of Supplementary Information Note 1. Comparisons of the predicted and observed *mean* increases in suspended sediment during the all of the plume experiments are shown in Figure S1-14 of Supplementary Information Note 1.

Table S2-3 Parameter settings used in modelling of plume experiments

| Time step (s) | 10 |
| --- | --- |
| Horizontal diffusion (m^2^/s) | 0.05 |
| Critical stress for deposition (N/m^2^) | 0.1 |
| Critical stress for erosion (N/m^2^) | 0.2 |
| Erosion rate parameter (kg/s/N) | 0.002 |
| Nikuradse roughness length (m) | 0.01 |
| Density of deposited material (kg/m^3^)  Fine particles (D < 63 µm)  Sand size particles (D > 63 µm) | 500  1600 |
| Height of plume release above bed | 2 m |
| Cross-sectional area of plume release (m^2^) | 0.0038 |
| Discharge of plume release (litres/s) | 5.0 |
| Release of different fractions in release | As Table S4 |
| Settling velocity of different sediment fractions | As shown in Table S4 |

Table S2-4 Release rates for different fractions plume experiment modelling

| **Fraction** | **Particle/floc diameter** | **Settling velocity** | **Release (kg/s)** |
| --- | --- | --- | --- |
| 1 | <63 | 0.004 mm/s | 0.0327 |
| 2 | 90 | As table 4 main text | 0.0110 |
| 3 | 125 | As table 4 main text | 0.0130 |
| 4 | 180 | As Soulsby (1997)* | 0.0225 |
| 5 | 250 |  | 0.0284 |
| 6 | 355 |  | 0.0489 |
| 7 | 500 |  | 0.0265 |
| 8 | 710 |  | 0.0093 |
| 9 | 1000 |  | 0.0040 |
| 10 | 1400 |  | 0.0016 |
| 11 | 2000 |  | 0.0012 |
| 12 | 2800 |  | 0.0007 |
| 13 | 4000 |  | 0.0004 |

*Settling velocity, $w_{s} ,$ is calculated using the formula $w_{s}=\frac{\nu}{d}\left[ \left( {10.36}^{2}+1.049D_{*}^{3} \right)^{1/2}-10.36 \right]$ where $D_{*}=\left[ \frac{g\left( s-1 \right)}{\upsilon^{2}} \right]^{1/3}d$ , and where $g$ is the acceleration due to gravity, $s$ is the specific gravity of the sediment, $\upsilon$ is the kinematic viscosity, and $d$ is the particle diameter. Soulsby, R.L. (1997) Dynamics of Marine Sands, Thomas Telford Publications, London.

- 1. **Description of plume modelling for experiments on 1 December**

The plume modelling for the experiments of **1 December** was undertaken using SEDPLUME –RW as above . The model used the hydrodynamic input from the TELEMAC run starting on 27 November and was run for the following experiments (Table S2-5):

Table S2-5 Description of modelled plume experiments for 1 December

| **Day** | **Plume Experiment** | **Experiment run** | **Time of start of experiment** | **Modelled period of release** |
| --- | --- | --- | --- | --- |
| 1 | 3 | 50 m from Lander, Run 1 | 03:55 | 10 mins |
| 1 | 3 | 50 m from Lander, Run 3 | 04:35:30 | 15 mins |
| 1 | 3 | 50 m from Lander, Moving source | 03:26 | 10½ mins |

The locations of the Lander and ROV source during these experiments are presented in Table S2-6.

Table S2-6 Locations of ROV source and Lander measurements during modelled plume experiments for 17 and 18 November

| **Day** | **Plume Experiment** | **Experiment run** | **Position (UTM 27)** | | | | |
| --- | --- | --- | --- | --- | --- | --- | --- |
|  |  |  | **ROV** | | | **Lander** | |
|  |  |  | **E (m)** | | **N (m)** | **E (m)** | **N (m)** |
| 1 | 3 | 50 m from Lander, Run 1 | 529429 | 2637905 | | 529442 | 2637857 |
| 1 | 3 | 50 m from Lander, Run 3 | 529425 | 2637901 | |  |  |
| 1 | 3 | 50 m from Lander, Moving source   - Start - End | 529421  529445 | 2637903  2637908 | |  |  |

All parameter settings were as for the modelling of the experiments on 17 and 18 November. Comparisons of the predicted and observed suspended sediment concentrations at the Lander are shown in Figures S1-11 to S1-13 of Supplementary Information Note 1. Comparisons of the predicted and observed *mean* increases in suspended sediment during the all of the plume experiments are shown in Figure S1-14 of Supplementary Information Note 1.

- 1. **Description of longer plume experiment simulation**

A longer simulation of the plume experiment of the 18 November , with release 100 m from the lander, starting at 19:39 was undertaken, this time lasting for 20,000 seconds (just under 6 hours).

- 1. **Description of flow and plume modelling for mining simulation**

The longer simulation above was repeated but this time assuming a continuous release arising from real-scale mining. The parameter settings used are summarised in Table S2-7 and the release rates for the different fractions are summarised in Table S2-8.

Table S2-7 Parameter settings used in modelling of plume experiments

| Time step (s) | 10 |
| --- | --- |
| Horizontal diffusion (m^2^/s) | 0.05 |
| Critical stress for deposition (N/m^2^) | 0.1 |
| Critical stress for erosion (N/m^2^) | 0.2 |
| Erosion rate parameter (kg/s/N) | 0.002 |
| Nikuradse roughness length (m) | 0.01 |
| Density of deposited material (kg/m^3^) | 500 |
| Release of different fractions in release | As Table S6 |
| Settling velocity of different sediment fractions | As Table S6 |

Table S2-8 Release rates for different fractions plume experiment modelling

| Fraction | Floc/particle diameter | Settling velocity (mm/s) | Release (kg/s) |
| --- | --- | --- | --- |
| 1 | <40 | 0.004 | 0.2 |
| 2 |  | 0.023 | 0.08 |
| 3 |  | 0.46 | 1.54 |
| 4 | 40-120 | As Table 4 main text | 1.66 |
| 5 | 120-160 | As Table 4 main text | 1.62 |
| 6 | > 160 | As Table 4 main text | 3.0 |

**Measurement and modelling of deep sea sediment plumes and implications for deep sea mining**

Jeremy Spearman, Jon Taylor, Neil Crossouard, Alan Cooper, Michael Turnbull, Andrew Manning, Mark Lee & Bramley Murton

**Supplementary Information part 3**

**Description of plume dispersion model**

**Contents**

- 1. Description of the SEDPLUME-RW far-field plume dispersion model
  2. Description of the SEDPLUME-RW near-field plume dispersion module
  3. References

##### **Description of the SEDPLUME-RW far-field plume dispersion model**

###### 3.1.1 Representation of sediment plume dispersion

In SEDPLUME-RW, the release of suspended sediment is represented as a regular or intermittent discharge of discrete particles. Particles are released throughout a model run to simulate continuous sediment disturbance or for part of the run to simulate sediment disturbance over an interval during the tidal cycle, for instance to represent the resuspension of fine sediment during dredging or mining operations. At specified sites a number of particles are released in each model time-step and, in order to simulate the release of suspended sediment, the total sediment released at each site during a given time interval is divided equally between the released particles. Particles can be released either at the precise coordinates of specified locations, or distributed randomly, centred on the specified release sites (which may move in time). The particles can be released at specific depths or intervals through the water column.

For more complicated cases the SEDPLUME-RW model makes use of an integral buoyant jet model to provide source terms for the far-field dispersion. This is described in Section 3.2.

###### Large scale advection

SEDPLUME-RW uses TELEMAC flow model results to provide hydrodynamic input. TELEMAC can be used to model either 2D or 3D currents. For TELEMAC-3D the 3D currents are used directly by SEDPLUME-RW.

Each particle is then advected by the local flow conditions. Because the three dimensional structure of the flow is calculated by SEDPLUME-RW, effects such as shear dispersion of plumes are automatically represented.

###### Turbulent diffusion

In order to simulate the effects of turbulent eddies on suspended sediment plumes in coastal waters, particles in SEDPLUME-RW are subjected to random displacements in addition to the ordered movements which represent advection by mean currents. The motion of simulated plumes is, therefore, a random walk, being the resultant of ordered and random movements. Provided the lengths of the turbulent displacements are correctly chosen, the random step procedure is analogous to the use of turbulent diffusivity in suspended sediment transport models. This is discussed in more detail below.

(a) Lateral diffusion

The horizontal random movement of each particle during a time-step of SEDPLUME consists of a displacement derived from the parameters of the simulation. The displacement of the particle in each of the orthogonal horizontal directions is calculated from a Gaussian distribution, with zero mean and a variance determined from the specified lateral diffusivity. The relationship between the standard deviation of the displacement, the time-step and the diffusivity is defined in Reference 1 as:

$\frac{\Delta^{2} }{\Delta t} = 2D$ (3)

where:

Δ = standard deviation of the turbulent lateral displacement (m)

Δt = time-step (s)

*D* = lateral diffusivity (m^2^s^-1^).

In a SEDPLUME-RW simulation, a lateral diffusivity is specified, which the model reduces to a turbulent displacement using Equation (3). No directional bias is required for the turbulent movements, as the effects of shear diffusion are effectively included through the calculated depth structure in the mean current profile.

(b) Vertical diffusion

Whilst lateral movements associated with turbulent eddies are satisfactorily represented by the specification of a constant diffusivity, vertical turbulent motions can vary significantly horizontally and over the water depth, so that vertical diffusivities must be computed from the characteristics of the mean flow field, rather than specified as constants. In neutral conditions, the vertical diffusivity, Kz, is given by (Reference 2):

$K_{z} = 0.16h^{2} \left(1-\frac{h}{d}\right)\frac{\partial u}{\partial z}$ (4)

where:

*h* = height of particle above the bed

*d* = water depth

0.16 = (von Karman constant)^2^

*u* = current speed

*z* = vertical coordinate

The value of the vertical diffusivity is calculated at each particle position, then a vertical turbulent displacement is derived for each particle from its *K_z_* value using an equation analogous to (3) for the lateral turbulent displacement.

In conditions of stratified density (salinity or sediment) the vertical diffusivity, Kz, is modified on the basis of the Richardson gradient number through the methodologies of Munk and Andersen (Reference 3) or Toorman (Reference 4).

(c) Drift velocities

A particle undergoes a random walk as follows:

$x^{n} = x^{n-1} +A(x^{n-1} ,t^{n-1} )\Delta t + B(x^{n-1} ,t^{n-1} )\sqrt{\Delta t} \xi^{n}$ (5)

where *x^n^* is the position of the particle at time *t^n^*, *A* is the advection velocity at timestep *n*-1 and *B* is a matrix giving the diffusivity. *ξ* is a vector of three random numbers, each drawn from a normal distribution with unit variance and zero mean. In the case of SEDPLUME-RW, *B* is diagonal, with the first two entries equal to √(2*D*) (as introduced in the previous section) and the third diagonal entry being equal to the local value of √(2*K_z_*) .

The movement of a particle undergoing a random walk as described in equation (5) can be described by the Fokker-Planck equation in the limit of a very large number of particles and a very short timestep, where we introduce subscripts *i*,*j* and *k* running over the three coordinate directions:

$\frac{\partial f}{\partial t} + \frac{\partial}{\partial x_{i} }(A_{i} f) = \frac{\partial^{2} }{\partial x_{i} \partial x_{j} }(\frac{1}{2}B_{ik} B_{jk} f)$ (6)

The probability density function f(x,t|x_0_,t_0_) is the probability of a particle which starts at position x_0_ at time t_0_ being at position *x* at time *t*.

Equation (6) can be compared with the advection-diffusion equation for the concentration of a pollutant, c:

$\frac{\partial c}{\partial t}+\frac{\partial}{\partial x_{i} }(u_{i} c) = \frac{\partial}{\partial x_{i} }(K_{ik} \frac{\partial}{\partial x_{k} }c)$ (7)

where K_ik_ is the eddy diffusion matrix, diagonal in our case but not necessarily so. Thus identifying f with c, we can see that the two equations are equivalent provided that we take the advection velocity as:

$A_{i} = u_{i} + \frac{\partial}{\partial x_{k} }K_{ik}$ (8)

In the case of SEDPLUME-RW, the diffusivity varies only in the vertical and is constant in the horizontal, so the horizontal advection velocity is simply the flow velocity (assuming that the relatively small effects of changing water depth can be neglected). However, when considering the movement of particles in the vertical it is important to include the gradient of the diffusivity (often referred to as a drift velocity) in the advection step. If this term is omitted then particles tend to accumulate in regions of low diffusivity, which in our case means at the surface and at the bed.

This subject is discussed in considerably more detail in References 5, 6, 7, and 8.

###### Sedimentation processes

(a) Settling

In SEDPLUME, the settling velocity (*w_s_*) of suspended sediment is assumed to be related to the suspended sediment concentration (*c*) through an equation of the form:

$w_{s} = \max(w_{\min} ,Pc^{Q} )$ (9)

where *w_min_*, *P* and *Q* are empirical constants. Having computed a suspended mud concentration field, as described subsequently in this section, a settling velocity can be computed in each output grid cell from Equation (7) and used to derive a downward displacement for each particle during each time-step of a model simulation. This displacement is added vectorially to the other computed ordered and random particle displacements. Note that there is a specified minimum value of w_s_. This results in settling velocities being constant at low suspended mud concentrations.

(b) Deposition

SEDPLUME-RW computes bed shear stresses from the input tidal flow fields using the rough turbulent equation, based on a bed roughness length input by the user. Where the computed bed stress, τ_b_, falls below a specified critical value, τ_d_, and the water is sufficiently deep, then deposition is assumed to occur. Mud deposition is represented in SEDPLUME by particles approaching the sea bed becoming inactive when τ_b_ is below τ_d_. Whilst active particles in the water column contribute to the computed suspended mud concentration field, as described subsequently in this appendix, inactive particles contribute to the mud deposit field.

(c) Erosion

The erosion of mud deposits from the sea bed is represented in SEDPLUME by inactive particles returning to the water column (becoming active) when τb exceeds a specified erosional shear strength, τ_e_. The number of particles which become re-suspended in each cell of the output grid in each time-step of a simulation is determined by the equation:

$\frac{\partial m_{e}}{\partial t}=M\left(\tau_{b}-\tau_{e}\right)$ (10)

where:

*m_e_* = the mass eroded (kg)

*t* = time (s)

*M* = an empirical erosion constant.

###### Computation of suspended mud concentrations

SEDPLUME-RW computes suspended mud concentrations are computed by summing the mass of mud represented by the particles in each 2D or 3D cell of the output grid, and assuming that the resulting mass is evenly distributed over the cell area.

###### Computation of mud deposit distributions

SEDPLUME-RW computes mud deposit distributions by summing the mass of mud represented by the inactive particles in each cell of the output grid, and assuming that the resulting mass is evenly distributed over the cell area.

##### **Description of the SEDPLUME-RW near-field plume dispersion module**

##### Introduction

The near-field plume module models the near-field mixing that occurs as the negatively buoyant jet of the overflow mixes with the surrounding waters.

##### Near-field mixing

The descent of the dynamic plume is reproduced using a Lagrangian technique whereby a thin disc (which can be thought of as a section of a bent cone) of the released dynamic plume is tracked as it moves under the forces of momentum and negative buoyancy. The technique has been used for both dredger plume and outfall plume modelling (e.g. References 9, 10 and 11).

Entrainment of ambient water into the plume is modelled using the formulations of Lee and Cheung (Reference 12) and accounts for both shear entrainment (i.e. as occurs in jets and dominates the initial stages of the dynamic descent) and forced entrainment (which dominates in the latter stages of descent and is due to the flow of ambient water into the plume). The increase in mass due to shear entrainment is given by:

| **** | (11) |
| --- | --- |

where,

|  with  | (12) |
| --- | --- |

and where Δ*M_s_* is the increase in plume mass due to shear entrainment, *F* is the local densimetric Froude number and is based on Schatzmann (References 12 and 13), $\varphi$ is the angle between the plume descent and the horizontal, *θ_k_* is the angle between the jet descent and the x-axis, *b_k_* is the radius of the dynamic plume element at time step *k*, *h_k_* is the thickness of the dynamic plume element, *ρ* is the density of the plume, *V_k_* is the speed of the dynamic plume element, *ρ_a_* is the density of the ambient sea water, Δρ is the difference in density between the plume and the ambient water, Δt is the time step, and *u'_amb_* is the current speed (in the coordinate system moving with the dredger) of the ambient fluid. The formula suits a wide range of flows and gives the correct buoyancy for a pure jet (no buoyancy just momentum) and a pure plume (no initial momentum, just a density difference) (Reference 11).

Lee and Cheung (Reference 11) also derived an equation for forced entrainment arising in a three dimensional trajectory. The resulting entrainment is as follows:

| $\Delta M_{k}=\rho_{a}u'_{amb}h_{k}b_{k}\left[2\sqrt{\sin^{2}\varphi_{k}+\sin^{2}\theta_{k}-(\sin\varphi_{k}\sin\theta_{k})^{2}}+\pi\frac{\Delta b_{k}}{\Delta s_{k}}\cos\varphi_{k}\cos\theta_{k}\right.$ | (13) | |
| --- | --- | --- |
| $\left.+\frac{\pi}{2}b_{k}\frac{(\cos\varphi_{k}\cos\theta_{k}-\cos\varphi_{k-1}\cos\theta_{k-1})}{\Delta s_{k}}\right]\Delta t$ |  |  |

where Δ*M_k_* is the increase in plume mass due to forced entrainment, *s_k_* is the distance travelled by dynamic plume in timestep *k*, and Δ*b_k_* is the change in plume radius, *b_k_*– *b_k_*_-1_ .

There are three different contributions to forced entrainment which are all significant at different times suggesting that none of these terms can be neglected. The first term represents the forced entrainment due to the projected area of the crossflow while the second and third terms represent corrections due to the growth of the plume radius and the curvature of the trajectory (References 11 and 14). The near-field descent phase is terminated either when the plume impinges on the bed, when the dynamic plume becomes sufficiently diffuse that it becomes a passive plume, or after a time set by the user.

###### **References**

1. H B Fischer, E J List, R C Y Koh, J Imberger and N H Brooks, 1979. Mixing in Inland and Coastal Waters. New York : Academic. 483 pp.
2. Nezu, I., and Nakagawa, H., Turbulence in open-channel flows, *IAHR Monograph Serie*s, Balkema, 1993.
3. Munk W H and Andersen E A (1948) Notes on a theory of the thermocline, J. Marine Research, 3(1): 276-295.
4. Toorman E A (2000) Parameterisation of turbulence damping in sediment-laden flows, Report HYD/ET/00/COSINUS3, Hydrualics Laboratory, Katholieke Univeriteit Leuven.
5. A S Monin and A m Yaglom. "Statistical Fluid Mechanics". MIT Press, Cambridge, Massachusetts, 1971.
6. A F B Tompson and L W Gelhar. "Numerical simulation of solute transport in three-dimensional randomly heterogeneous porous media". Water Resources Research, Vol 26 pp2541-2562, October 1990.
7. K N Dimou and E E Adams. "A random-walk particle tracking model for well-mixed estuaries and coastal waters". Estuarine, Coastal and Shelf Science, Vol 37, pp99-110, 1993.
8. B J Legg and M R Raupach. "Markov-chain simulation of particle dispersion in inhomogeneous flows: the mean drift velocity induced in a gradient in Eulerian velocity variance". Boundary Layer Meteorology Vol 24, pp3-13, 1982.

Koh R C Y and Chang Y C , 1973, Mathematical model for barged ocean disposal of waste, Technical Series EPA 660/2-73-029, US Environment Protection Agency, Washington D.C.

Brandsma M G and Divoky D J, 1976, Development of models for prediction of short-term fate of dredged material discharged in the estuarine environment, Contract Report D-76-5, US Army Engineer Waterways Experiment Station, Vicksburg, MS, prepared by Tetra Tech, Inc., Pasadena, CA.

Lee J H W and Cheung V, 1990, Generalized lagrangian model for buoyant jets in current, Journal of Environmental Engineering, volume 116, number 6.

Schatzmann M, 1979, Calculation of submerged thermal plumes discharged into air and water flows, Proceedings of the 18th IAHR Congress, International Association for Hydraulic Research, Delft, 4, pp379-385.

Schatzmann , 1981, Mathematical modelling of submerged discharges into coastal waters, Proceedings of the 19th IAHR Congress, International Association for Hydraulic Research, Delft, 3, pp239-246.

Frick W E, 1984, Non-empirical closure of the plume equations, Atmospheric Environment, Volume 18, Number 4, 653-662.
